# Supplementary material for: DDX3X overexpression in mice can cause rapid tissue-specific toxicity and mortality
Source: Mol Ther. 2026 Jul 15;34(9):5135–44. doi: 10.1016/j.ymthe.2026.07.032 (PMC13555558; doi:10.1016/j.ymthe.2026.07.032)
Supplement: Supplementary file 1 [file mmc1.pdf]

YMTHE, Volume *34*

Supplemental Information

DDX3X overexpression in mice can cause rapid tissue-specific toxicity and mortality

Andrea Boitnott, Yuhui Hu, Mary Wight-Carter, Christian Lopez Escobar, Xin Chen, and Steven J. Gray

Supplemental Material

Mice

In addition to the C57BL/6J wild-type mice, a novel loss-of-function DDX3X mouse line was generated by the Transgenic Technology Center Core at UT Southwestern Medical Center. This mouse was used for the RT-qPCR experiments to assess gene expression. It was designed to knock-in an allele containing an invertible stop cassette, configured as a flip-excision (FLEx) switch (FRT-LoxP-Lox2272-inverted SV40pA x3-LoxP-Lox2272-FRT), inserted into *Ddx3x* intron 1. Correct editing was confirmed in the founder by whole-genome sequencing. Mice were bred to obtain *Ddx3x*$^{FLEx/FLEx}$ females, which were then crossed with Sox2-cre/+ males (#008454; The Jackson Laboratory) to generate *Ddx3x*$^{FLEx/+}$ control, referred to as wild-type, and *Ddx3x*$^{stop/+}$ mutant, referred to as *Ddx3x*$^{+/-}$, female littermates. This line was maintained on a C57BL6/J background. Stop cassette efficiency was validated by DDX3X protein quantification via western blot from cortical tissue (data not shown). Additionally, *Ddx3x*$^{+/-}$ females exhibit a phenotype reminiscent of a previously characterized haploinsufficient *Ddx3x* mouse model and of DDX3X syndrome, supporting the relevance and validity of this model (data not shown).

Viral Vector Injections

For bilateral ICV injections, pups were cryoanesthetized and moved to a precooled clay mold for stabilization. A stereotaxic injection syringe (65460-06, Hamilton) with a 33 gauge needle (65461-02, Hamilton) set to a stop depth of 2 mm was used and needle placement was determined based on lambda, the midline, and the eye, such that if a line was drawn from lambda to the eye, the placement would be 1 mm from lambda towards the eye and about 0.5 mm from midline. A light was used to help visualize the skull suture lines under the skin. 1 µL was injected over 1 minute per hemisphere, with a subsequent 30 second hold before withdrawing the needle. Pups were paw tattooed for identification and placed on a heating pad until fully conscious before returning to their home cage.

Tissue Collection

Pups were cryoanesthetized, whereas adult mice were anesthetized with 500 mg/kg 2,2,2-tribromoethanol (421430500, Thermo Scientific Chemicals) dissolved in 2-methyl-2-butanol (152463, Sigma-Aldrich) and diluted in sterile water to a final concentration of 2.5%, prepared fresh on the day of use. Following full anesthesia, the chest cavity was opened and blood was collected by cardiac puncture using a 1 mL syringe fitted with a 25 gauge needle. The mice were then perfused with 1x PBS and 1 U/mL heparin at a flow rate of 1 mL/minute for pups and 3.5 mL/minute for adults. The color of the liver was noted before and after perfusion for necropsies on P6. Heart, liver, lung, kidney, and brain tissue were collected in three ways for downstream processing. (1) Frozen tissue: the outer half of the brain left hemisphere, bottom third or heart, one lobe of the liver, and half of the right kidney cut longitudinally was frozen on dry ice and stored at -80°C. (2) Formalin fixed tissue: the inner half of the brain left hemisphere, upper third of the heart, one lobe of the liver, and half of the right kidney cut longitudinally were fixed in 10% formalin for 24 hours at room temperature. Following fixation, the samples were washed in 70% ethanol three times and then secured in cassettes for paraffin embedding. (3) Paraformaldehyde (PFA) fixed tissue: the brain right hemisphere, middle third of the heart, one lobe of liver, and left kidney were fixed in 4% PFA for 24

hours at 4°C. The samples were then rinsed in Milli-Q water three times, transferred to 10% sucrose + 0.5% sodium azide for 24 hours at 4°C, followed by 18% sucrose + 0.05% sodium azide for 48 hours at 4°C. For brain, an additional sucrose step of 30% sucrose + 0.05% sodium azide for 48 hours at 4°C was done. Following the sucrose gradient, the samples were embedded in optimal cutting temperature (OCT) compound.

Table S1. Pathology report provided by a veterinary pathologist with toxicity findings from H&E and ORO slides, including mouse ID, sex, treatment, route of administration, age at necropsy, and tissue type.

Table S2. Forward and reverse primer sequences used for RT-qPCR.

**Category**	**Gene**	**Forward Primer**	**Reverse Primer**
Gene of Interest	Mouse DEAD-box helicase 3 X-linked (*Ddx3x*)	ATGAGTCATGTGGCAGTGGA	CCTTTGCTTGCTGTACTTCC
	Human *DDX3X* opt	CGAAGATGACTGGAGCAAGC	AATTGTTTCCGGTGGCTTCC
Housekeeping normalization	Ribosomal protein L13a (*Rpl13a*)	CTGCTCTCAAGGTTGTTCGGCT	CCTTCCGTTTCTCCTCCAGAGT
Lipid metabolism and transport	Apolipoprotein B (*ApoB*)	GCATGAGTATGCCAATGGTCTCC	CTGGTTGCCATCTGAAGCCATG
	CD36 molecule (*Cd36*)	GGACATTGAGATTCTTTTCCTCTG	GCAAAGGCATTGGCTGGAAGAAC
	Perilipin 2 (*Plin2*)	GACAGGATGGAGGAAAGACTGC	GGTAGTCGTCACCACATCCTTC
	Carnitine palmitoyltransferase 1A (*Cpt1a*)	GGCATAAACGCAGAGCATTCCTG	CAGTGTCCATCCTCTGAGTAGC
	Peroxisome proliferator activated receptor alpha (*Pparα*)	ACCACTACGGAGTTCACGCATG	GAATCTTGCAGCTCCGATCACAC
Type I interferon-driven innate immune response	DExD/H-box helicase 58 (*Ddx58*; RIG-I)	AGCCAAGGATGTCTCCGAGGAA	ACACTGAGCACGCTTTGTGGAC
	Interferon beta 1 (*Ifnb1*)	GCCTTTGCCATCCAAGAGATGC	ACACTGTCTGCTGGTGGAGTTC
	C-X-C motif chemokine ligand 10 (*Cxcl10*)	ATCATCCCTGCGAGCCTATCCT	GACCTTTTTTGGCTAAACGCTTTC
Cell stress and death	DNA damage inducible transcript 3 (*Ddit3*; CHOP)	GGAGGTCCTGTCCTCAGATGAA	GCTCCTCTGTCAGCCAAGCTAG
	BCL2 interacting protein 3 (*Bnip3*)	GCTCCAAGAGTTCTCACTGTGAC	GTTTTTCTCGCCAAAGCTGTGGC

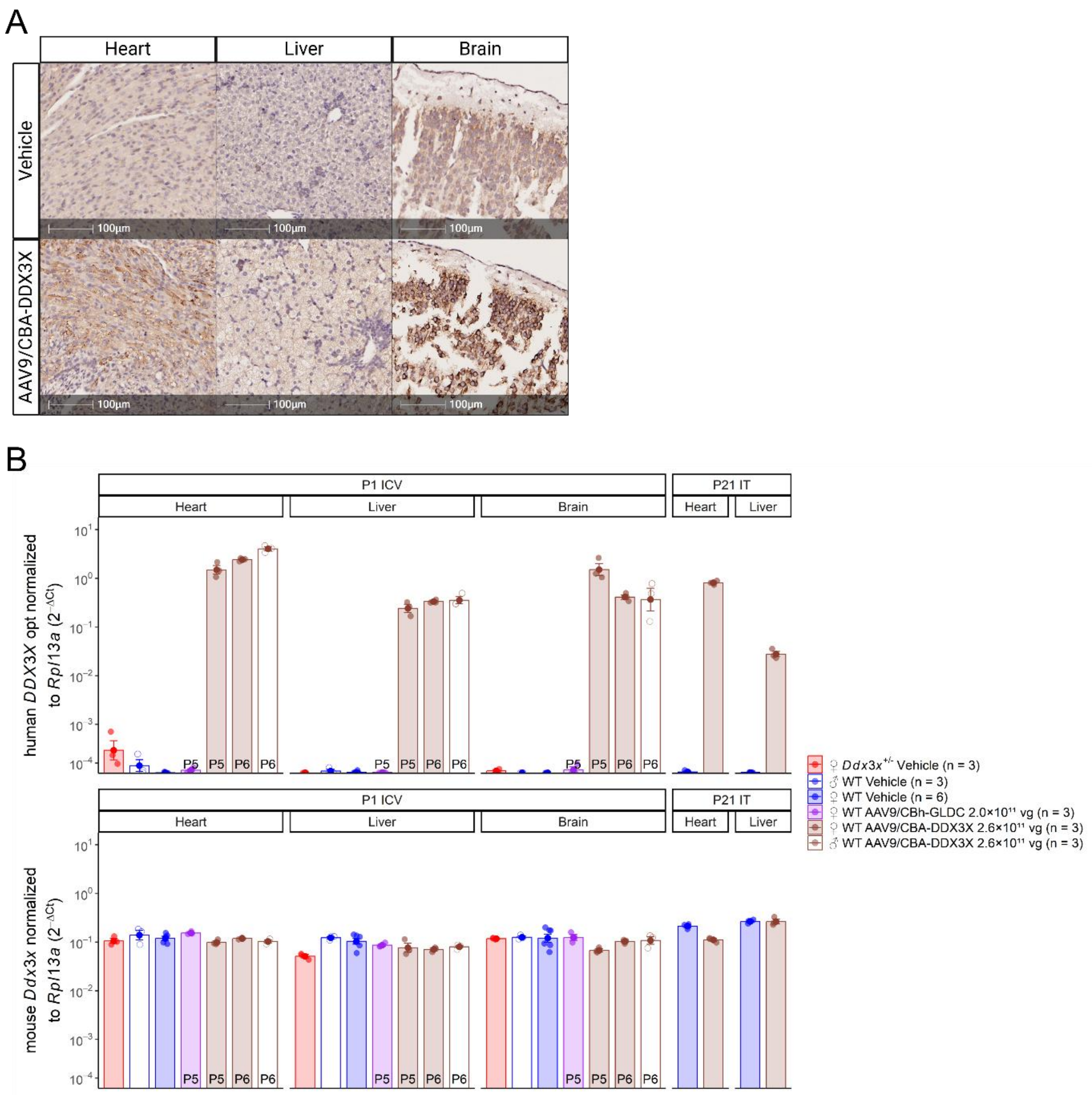

Figure S1. A) DDX3X IHC staining of heart, liver, and brain sections scanned at 20x magnification from representative wild-type mice on P6 following a P1 ICV injection of vehicle or AAV9/CBA-DDX3X (2.6 x 10^{11} vg). Scale bars = 100 µm. Quantification from all mice in Figure 1B. B) Human *DDX3X* opt (top) and endogenous mouse *Ddx3x* (bottom) mRNA expression in the heart, liver, and brain from female and male mice injected with vehicle or AAV9/CBA-DDX3X (2.6 x 10^{11} vg) on P1 or P21 via direct CSF injection. Values are defined as gene expression normalized to the housekeeping gene *Rpl13a*, calculated as $2^{-\Delta Ct}$, where ΔCt = Ct(Ddx3x) – Ct(Rpl13a). A subset is presented in Figure 1B (middle and bottom panels).

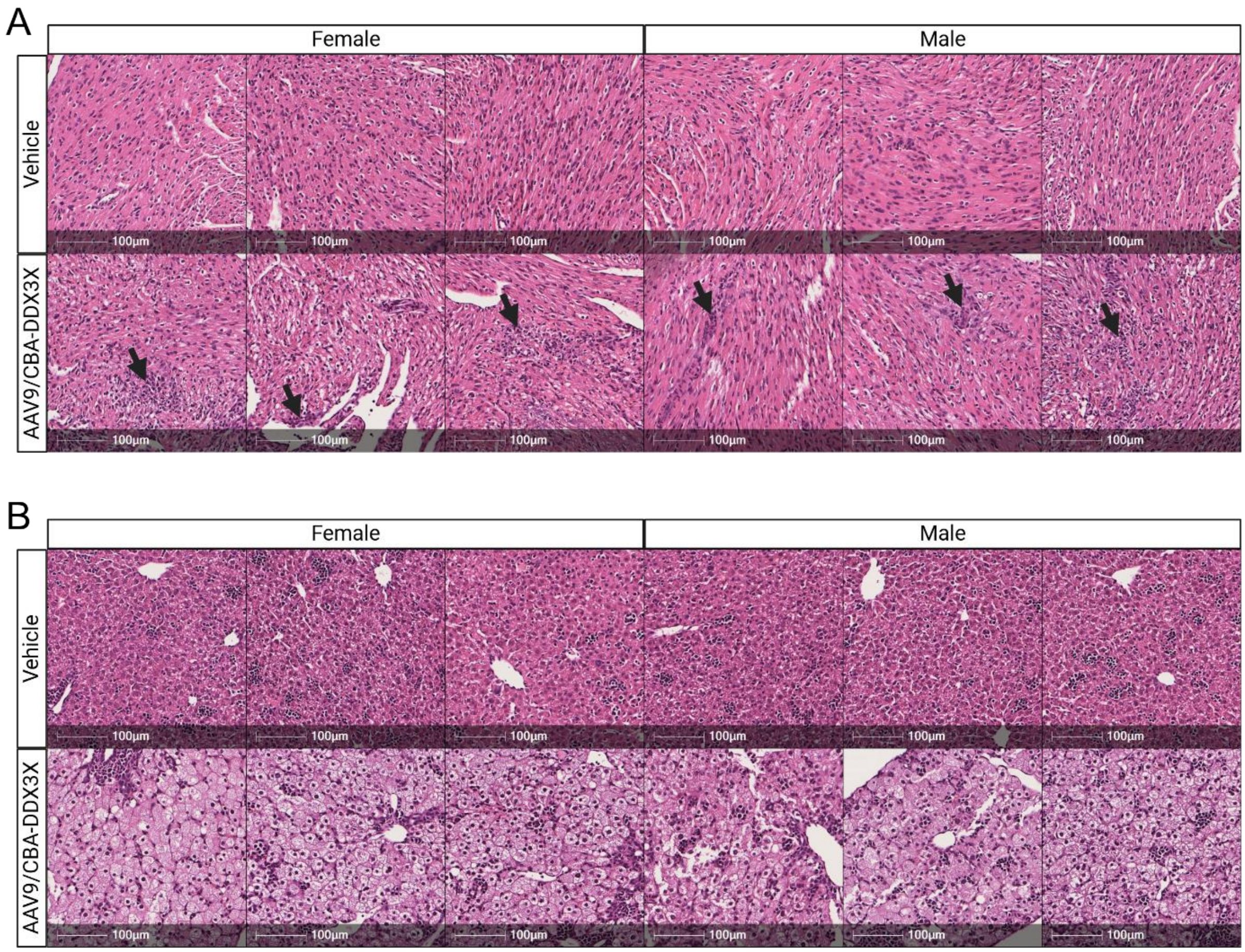

Figure S2. A) H&E staining of heart sections scanned at 20x magnification from wild-type mice on P6 following a P1 ICV injection of vehicle or AAV9/CBA-DDX3X (2.6 x 10^{11} vg). Three biological replicates for each group are shown. Arrows indicate example areas of myocardial degeneration. Scale bars = 100 µm. B) H&E staining of liver sections scanned at 20x magnification from the same mice in A. Column 1 from A and B are used as the representative image in Figure 1C. Scale bars = 100 µm.

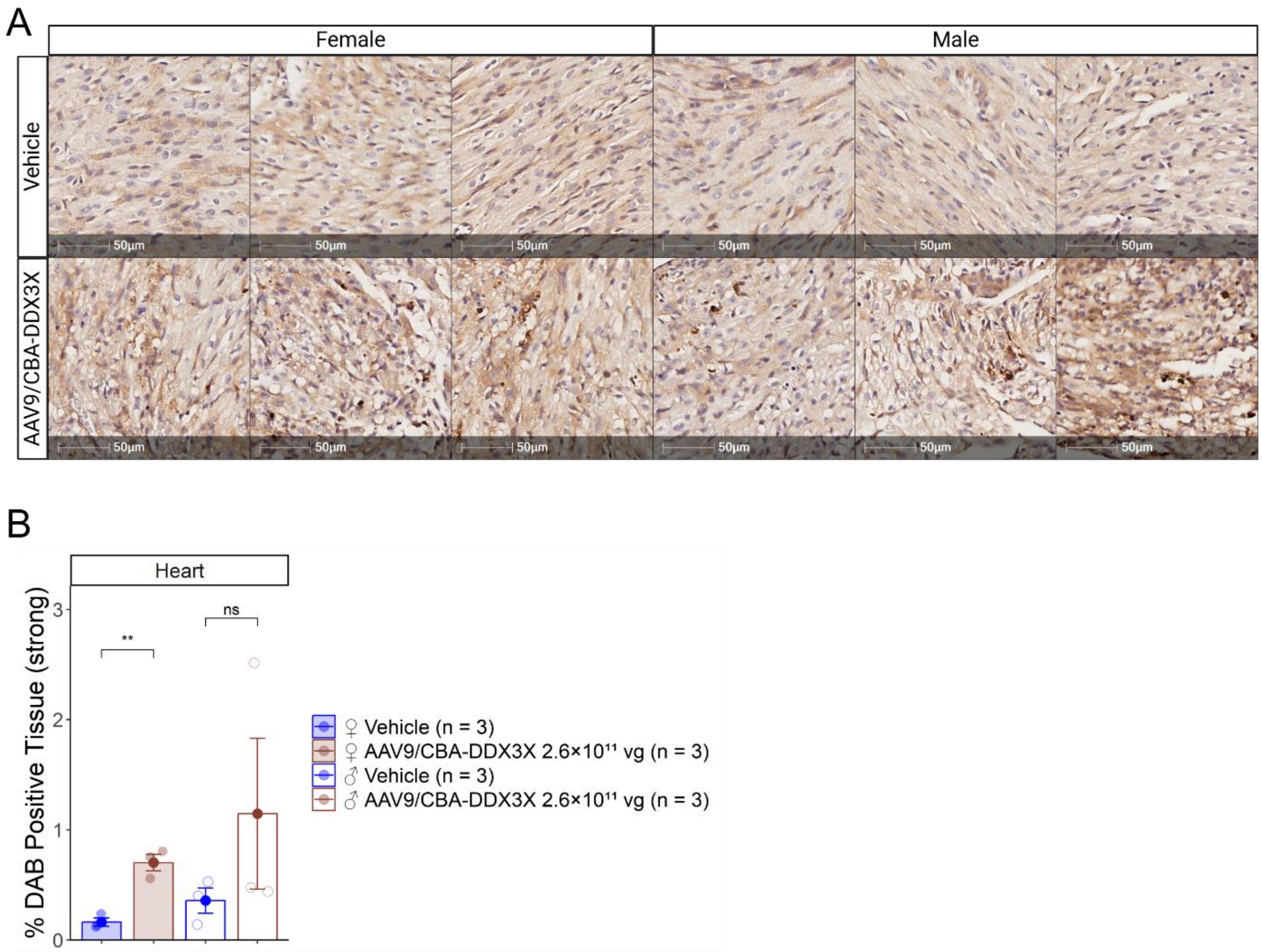

Figure S3. A) Cleaved caspase 3 IHC staining of heart sections scanned at 40x magnification from wild-type mice on P6 following a P1 ICV injection of vehicle or AAV9/CBA-DDX3X (2.6 x 10^{11} vg). Three biological replicates for each group are shown. Column 1 is used as the representative image in Figure 1F. Scale bars = 50 µm. B) Cleaved caspase 3 IHC quantification: Percentage of strong DAB positive stain area in heart sections from the mice with representative images in A. Statistical comparisons between groups were calculated using pairwise Welch's t-tests.

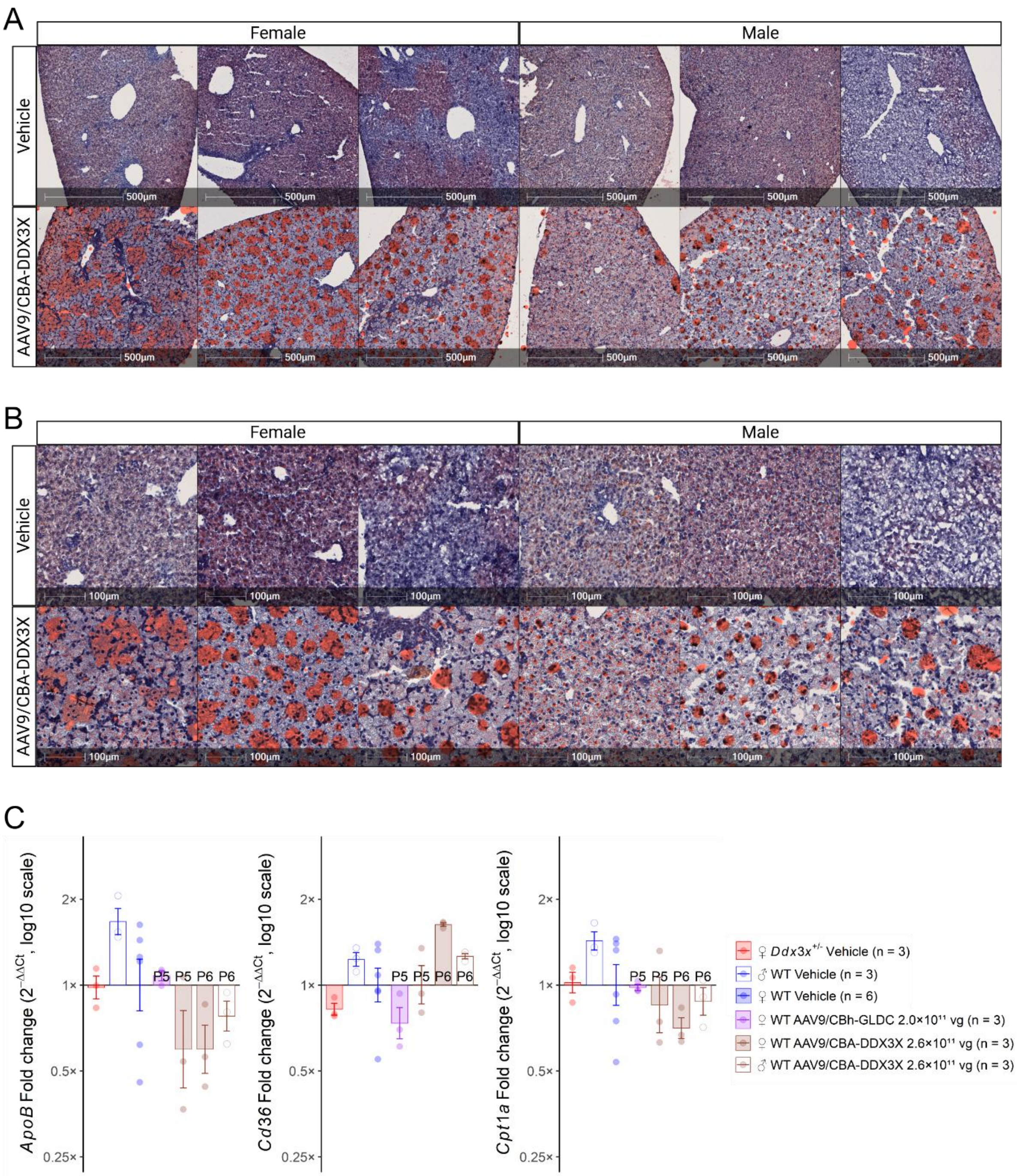

Figure S4. A) ORO staining of liver sections scanned at 7.5x magnification from wild-type mice on P6 following a P1 ICV injection of vehicle or AAV9/CBA-DDX3X (2.6 x 10^{11} vg). Three biological replicates for each group are shown. Scale bars = 500 µm. B) 20x magnification of the sections in A. Column 1 is used as the representative image in Figure 2B. Scale bars = 100 µm. C) Relative *ApoB* (left), *Cd36* (middle), and *Cpt1a* (right) mRNA expression in the liver from female and male mice injected with vehicle or AAV9/CBA-DDX3X (2.6 x 10^{11} vg) on P1 via ICV administration. Relative expression differences were assessed by pairwise Welch's t-tests on log2FC for each group

compared to the female wild-type vehicle group with Benjamini-Hochberg adjustment for multiple testing – no comparison was significant.

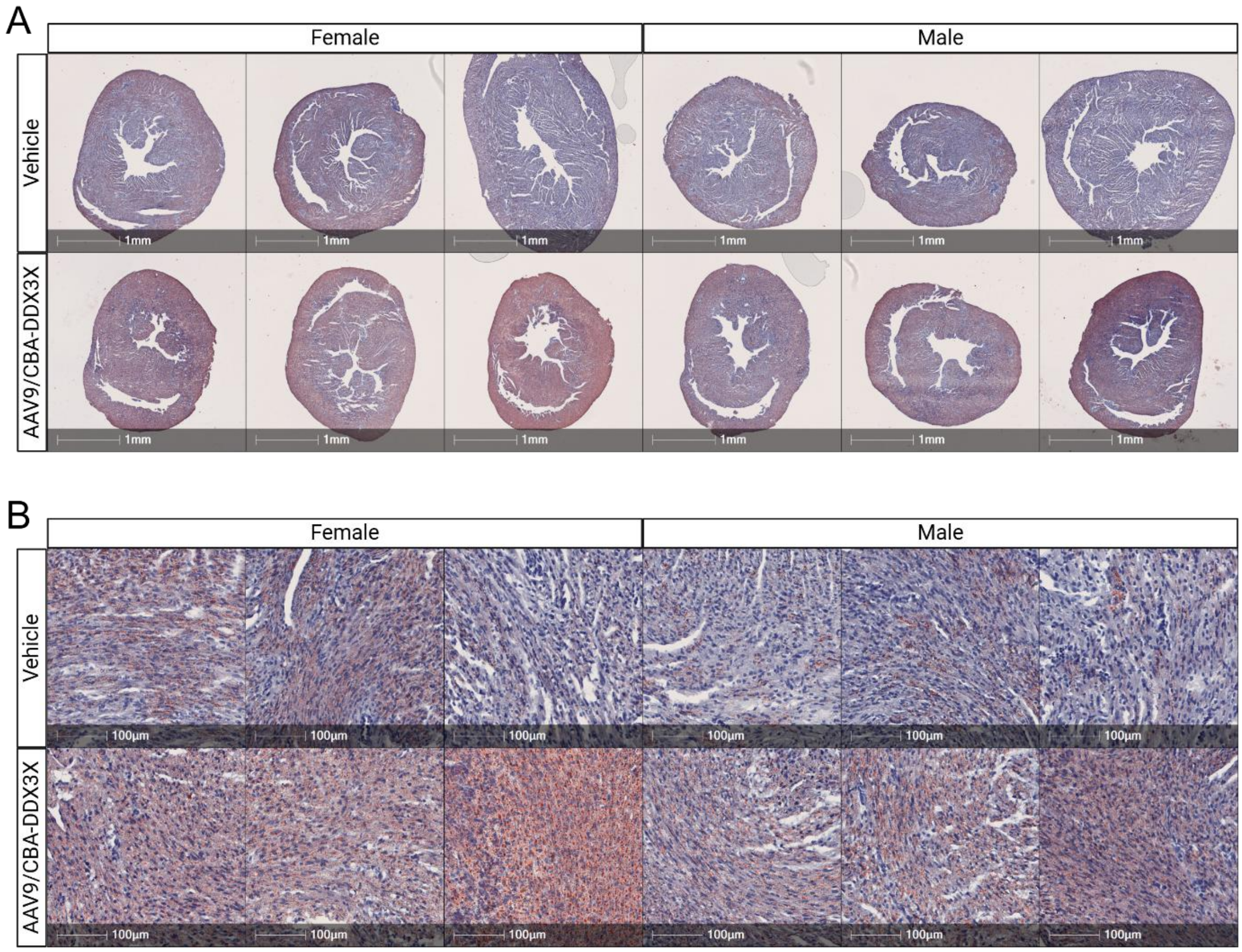

Figure S5. A) ORO staining of heart sections scanned at 2.5x magnification from wild-type mice on P6 following a P1 ICV injection of vehicle or AAV9/CBA-DDX3X (2.6 x 10^{11} vg). Three biological replicates for each group are shown. Scale bars = 1 mm. B) 20x magnification of the sections in A. Column 1 is used as the representative image in Figure 2B. Scale bars = 100 µm.

UTSouthwestern
Medical Center

Translational Gene Therapy Core (TGTC)

Certificate of Analysis Form

**Batch ID:**	NC-56-23-001_02
**Product Name:**	pAAV9 / DDX3X
**ITR Plasmid Name:**	pTRsk-CBA-DDX3X-spA
**Manufacturing Date:**	13JUN2023
**Manufacturing Scale**	7.5L (of 10L)
**Vial Number:**	13
**Total Volume:**	0.46mL (10ulx5vials, 50ulx7vials, 60ulx1vial)
**Storage Conditions:**	≤-60°C, Upon thawing, 4°C

Test	Method	Results
Endotoxin	LAL	<1.00 EU/mL
Capsid Identity	ELISA	1.09E+15 Vp/mL
Capsid Identity	Stunner	6.50E+14 Vp/mL
Titer-Vg	qPCR, target ITR	1.78E+14 Vg/mL
Titer-Vg	Stunner	1.44E+14 Vg/mL
Purity	Silver Stain	Detection of VP1, VP2 and VP3 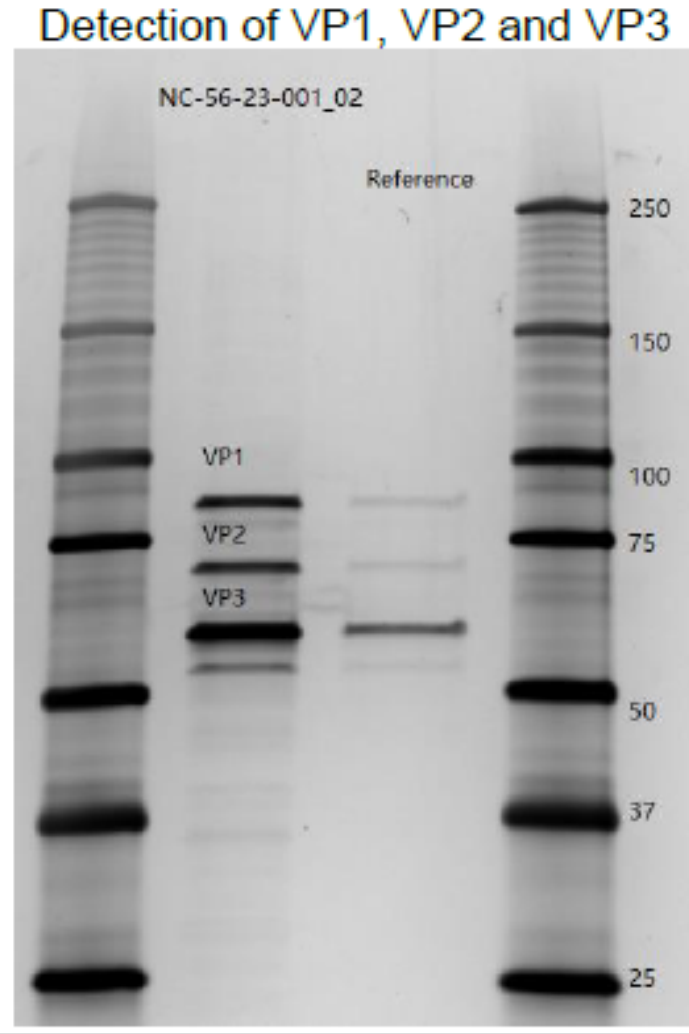

Released By Yang Yu

Digitally signed by Yang Yu
DN: cn=Yang Yu, c=US, o=UTSW, ou=VVC, email=yang.yu@utsouthwestern.edu
Reason: I am the author of this document
Date: 2023.06.16 16:31:51 -05'00'

Figure S6. The certificate of analysis summarizing quality control testing for the manufacture and release of AAV9/CBA-DDX3X from the Translational Gene Therapy Core at UT Southwestern Medical Center. The same batch was used for all injections.

UNIVERSITY OF NORTH CAROLINA VECTOR CORE

Quality Control Summary

Lot #	LAV117-conc	Name	GLDC

Test by qPCR

Test #	Titer, vg/mL	Analyst	Date	File
1	1.03E+14	PZ	01/29/2020	20200129-1147-gh-pz

PAGE analysis

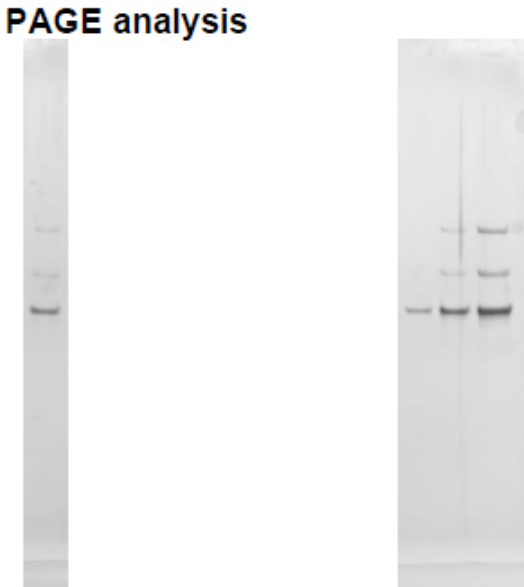

Loaded 5.00E+09 vg 4980E std 2e9vg 5e9vg 1e10vg
Calculated 4.10E+09 vg

Analyst	Ping Zhang
Date	01/13/2020
Reference #	20200113-silver

SEM

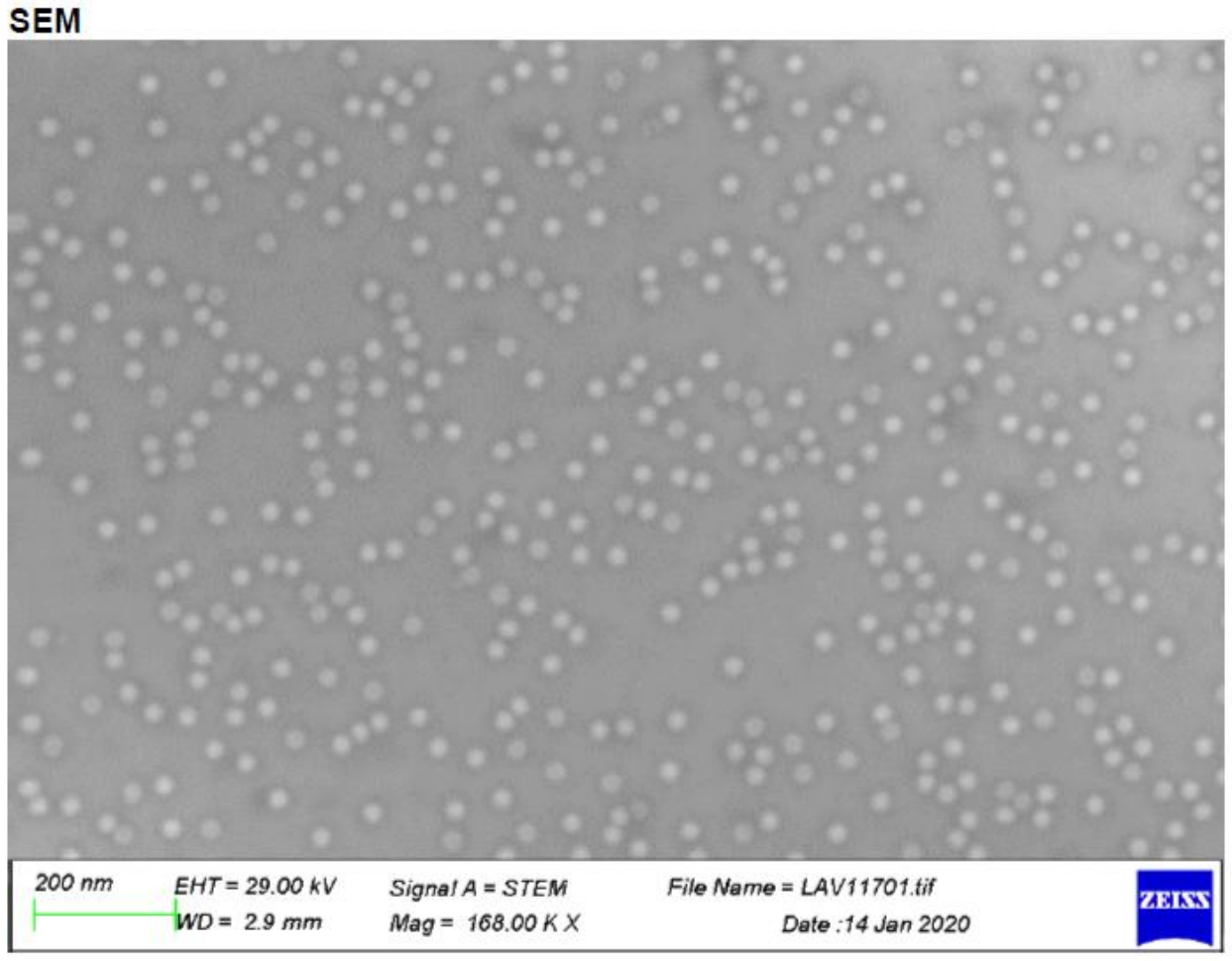

94% full

Analyst	Ping Zhang
Date	12/16/2019
Reference #	20191216-LAV117-01

Figure S7. The certificate of analysis summarizing quality control testing for the manufacture and release of AAV9/CBh-GLDC from the Vector Core at University of North Carolina. The same batch was used for all injections.

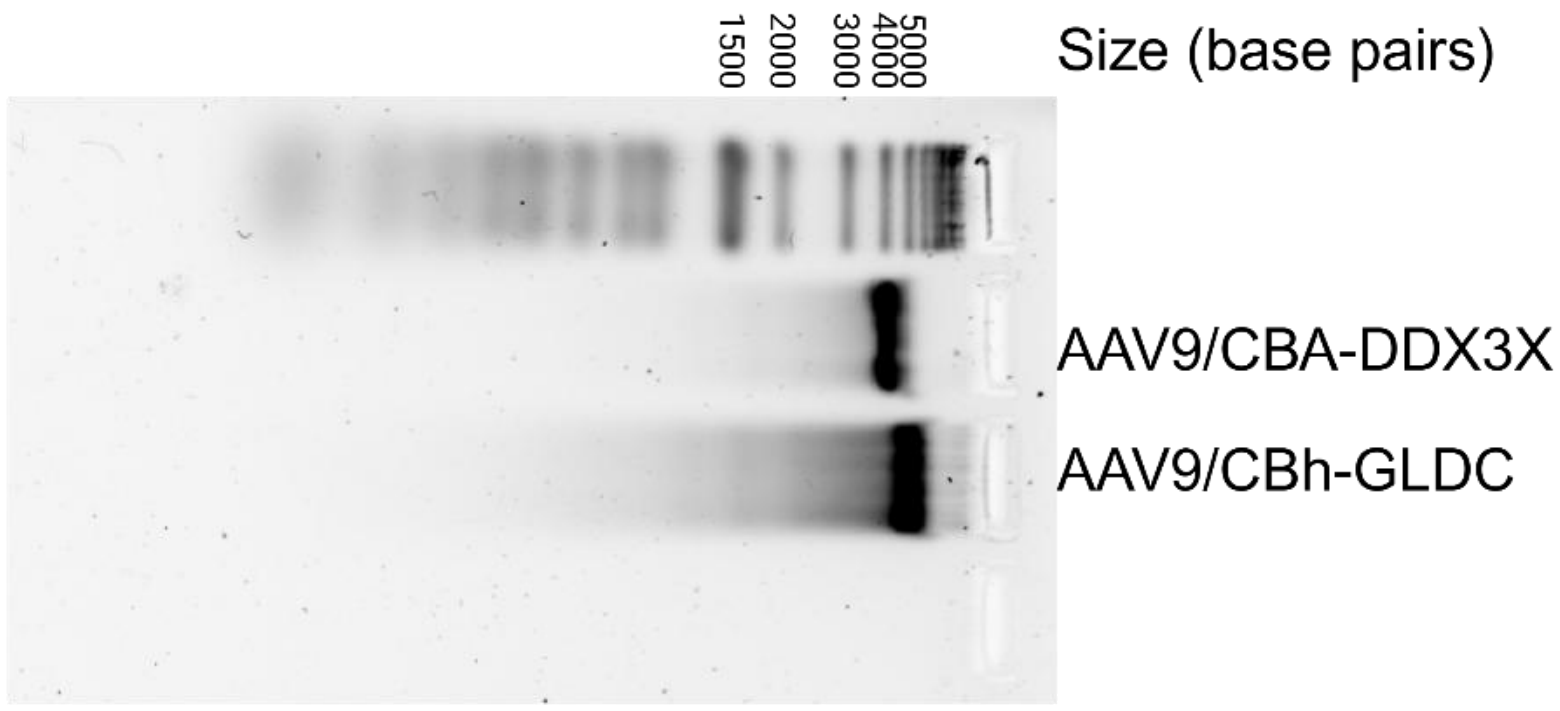

Figure S8. Alkaline gel showing viral vector integrity of AAV9/CBA-DDX3X and AAV9/CBh-GLDC at 5 x 10^{10} vg.
