# Supplementary material for: DDX3X overexpression in mice can cause rapid tissue-specific toxicity and mortality
Source: Mol Ther. 2026 Jul 15;34(9):5135–44. doi: 10.1016/j.ymthe.2026.07.032 (PMC13555558; doi:10.1016/j.ymthe.2026.07.032)
Supplement: Supplementary file 2 [file mmc3.pdf]

Molecular Therapy
Brief report

DDX3X overexpression in mice can cause rapid tissue-specific toxicity and mortality

Andrea Boitnott,[1] Yuhui Hu,[1] Mary Wight-Carter,[2] Christian Lopez Escobar,[3] Xin Chen,[1] and Steven J. Gray[1]

[1]Department of Pediatrics, UT Southwestern Medical Center, Dallas, TX 75390, USA; [2]Animal Resource Center, UT Southwestern Medical Center, Dallas, TX 75390, USA; [3]Histopathology Core, UT Southwestern Medical Center, Dallas, TX 75390, USA

DEAD-Box Helicase 3 X-Linked (DDX3X) is a ubiquitously expressed RNA helicase with diverse cellular roles implicated in a neurodevelopmental disorder called DDX3X syndrome. Although DDX3X is a leading genetic cause of intellectual disability in females, there is no treatment. While gene supplementation is a plausible therapeutic strategy, previous studies suggest DDX3X is carefully regulated and dose sensitive. To understand the consequences of overexpressing DDX3X with unregulated adeno-associated virus-mediated gene supplementation, we generated a vector driving strong ubiquitous DDX3X expression and administered it through a direct cerebrospinal fluid injection in newborn mice. Mice injected with a high dose died within 1 week from myocardial degeneration. Increased expression of stress response markers together with elevated apoptotic signaling in the heart suggested activation of stress-induced apoptotic pathways. Incidental findings included excess lipid accumulation, most prominent in the liver, and other liver injury. The innate immune system was also highly activated in the heart and liver. Interestingly, the brain was overall unaffected. The results suggest that DDX3X overexpression can cause rapid transgene-driven, tissue-specific toxicity, underscoring the need for tight *DDX3X* gene dosage control. These findings illustrate the possibility for improper transgene expression to drive severe toxicity including death within days following administration.

INTRODUCTION

DEAD-Box Helicase 3 X-Linked (DDX3X) is a ubiquitously expressed RNA helicase best known for its role in RNA metabolism, regulating transcription, translation, and RNA export.[1,2] Its multifunctional profile translates into broad effects on cellular programs, including signal transduction, cellular stress response, programmed cell death, embryonic development, and innate immune responses. Importantly, the role of DDX3X in these processes is highly complex and context dependent. For example, depending on cell type, tissue, and cellular state, DDX3X can inhibit or promote programmed cell death pathways, including apoptosis and pyroptosis.[3,4] Similarly, DDX3X can either amplify or restrain innate immune signaling depending on cell type, DDX3X localization, and whether the trigger is self or non-self.[5]

With a wide range of functions and roles, DDX3X has been implicated in a variety of human diseases, including a neurodevelopmental disorder called DDX3X syndrome.[6] Most patients with DDX3X syndrome are female with *de novo* loss-of-function variants in *DDX3X* that result in a spectrum of clinical features, including mild to severe intellectual disability, motor impairments, sleep disturbances, and other comorbid conditions like structural heart abnormalities.[7] There is no treatment. Instead, symptoms are managed with physical, occupational, behavioral, and speech therapy along with medications. Previously, our lab explored a DDX3X gene supplementation therapy approach in a haploinsufficient *Ddx3x* mouse model. For this approach, an adeno-associated virus serotype 9 (AAV9) construct with a weak ubiquitous promoter, JeT, was used to drive low levels of DDX3X. However, efficacy was limited, and phenotypes observed in haploinsufficient *Ddx3x* mice, like anxiety-like behavior, could be induced in wild-type mice treated with the vector.[8] The results suggested that DDX3X expression is tightly regulated, with both too little and too much DDX3X causing abnormal and negative consequences.

The concern of DDX3X overexpression and unregulated expression causing negative outcomes is critical to delineate when considering a one-time gene therapy for patients, as there is currently no standardized option to turn the transgene cassette off after administration. To understand the effect of overexpressing DDX3X, we generated an AAV9-mediated gene supplementation vector that expressed DDX3X at high levels ubiquitously with the synthetic chicken β-actin (CBA) promoter. This promoter drives much stronger transgene expression than the synthetic JeT promoter used in our previous study. We hypothesized that overexpressing DDX3X would cause negative consequences in mice. Our results were surprising in that transgene toxicity was rapid, causing lethality within 1 week of high-dose administration, and that the organ systems primarily involved with this toxicity were unrelated to the central nervous system (CNS) even though a direct cerebrospinal fluid (CSF) injection was used.

Received 16 February 2026; accepted 13 July 2026;
https://doi.org/10.1016/j.ymthe.2026.07.032.

Correspondence: Steven J. Gray, Department of Pediatrics, UT Southwestern Medical Center, Dallas, TX 75390, USA.
E-mail: steven.gray@utsouthwestern.edu

Published by Elsevier Inc. on behalf of The American Society of Gene and Cell Therapy.
This is an open access article under the CC BY license (http://creativecommons.org/licenses/by/4.0/).

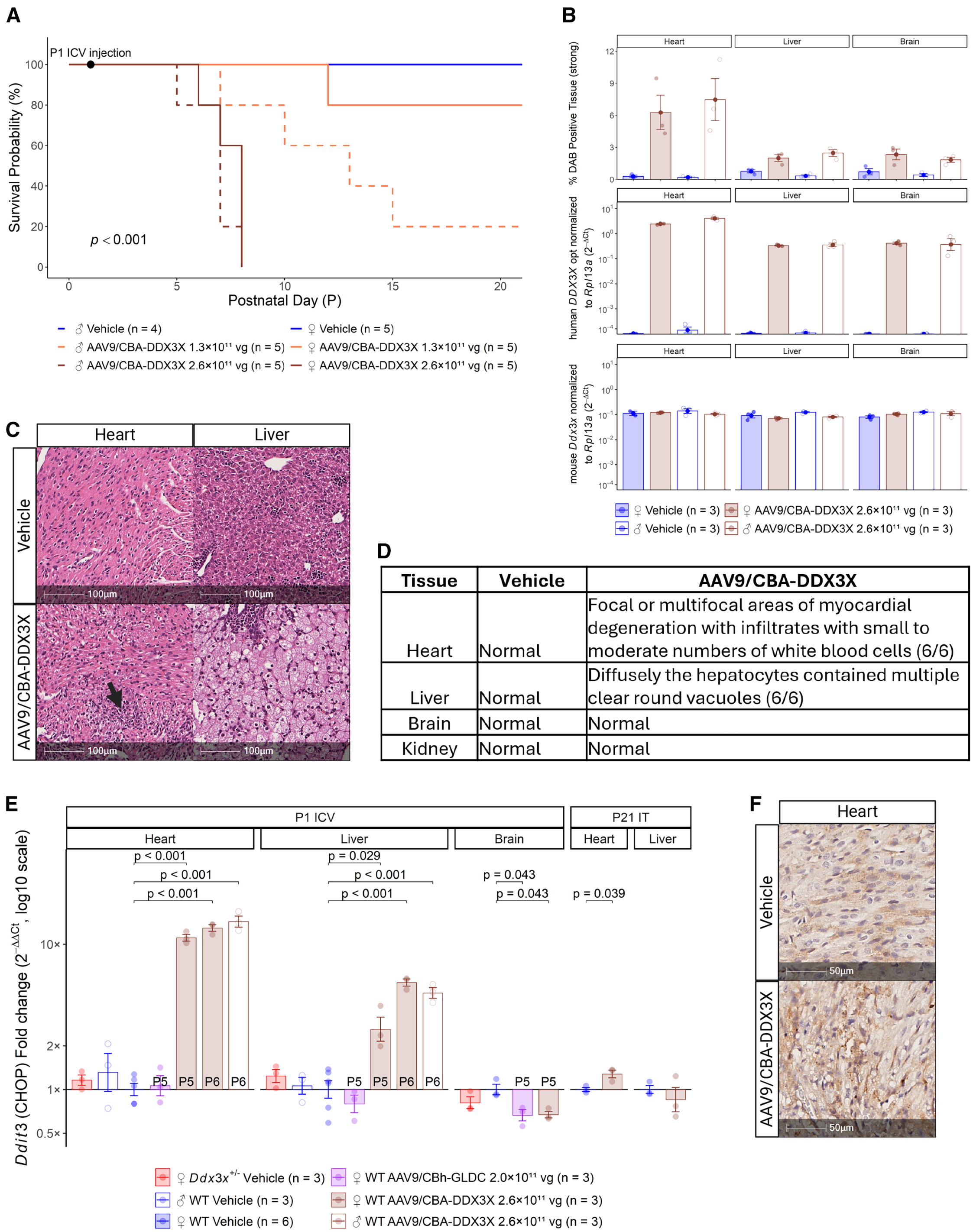

Tissue	Vehicle	AAV9/CBA-DDX3X
Heart	Normal	Focal or multifocal areas of myocardial degeneration with infiltrates with small to moderate numbers of white blood cells (6/6)
Liver	Normal	Diffusely the hepatocytes contained multiple clear round vacuoles (6/6)
Brain	Normal	Normal
Kidney	Normal	Normal

Figure 1. Overexpressing DDX3X can cause rapid death due to myocardial degeneration

(A) Survival probability of male and female wild-type mice injected with vehicle or AAV9/CBA-DDX3X (1.3×10^{11} or 2.6×10^{11} vg) on P1 via i.c.v. administration. Survival distributions were compared across groups using the Mantel-Cox log rank test, and the reported *p* value reflects the global comparison among all groups. (B) DDX3X IHC quantification (top), showing the percentage of strong DAB-positive stain area in heart, liver, and whole brain sections from male and female wild-type mice on P6 following an i.c.v. injection of vehicle or AAV9/CBA-DDX3X (2.6×10^{11} vg) on P1. Human *DDX3X* opt (middle) and endogenous mouse *Ddx3x* (bottom) mRNA expression in the heart, liver, and brain from male and female wild-type mice on P6 following an i.c.v. injection of vehicle or AAV9.CBA-DDX3X (2.6×10^{11} vg) on P1. Values are defined as gene expression normalized to the housekeeping gene *Rpl13a*, calculated as $2^{-\Delta Ct}$, where $\Delta Ct = Ct(Ddx3x) - Ct(Rpl13a)$. Error bars represent standard error of the mean. (C) H&E

(legend continued on next page)

RESULTS

Overexpressing DDX3X can cause rapid death due to myocardial degeneration

To determine the effect of overexpressing DDX3X, male and female wild-type mice were injected with vehicle or AAV9/CBA-DDX3X at 1.3×10^{11} or 2.6×10^{11} vg on postnatal day (P) 1 via bilateral intracerebroventricular (i.c.v.) administration. The doses are consistent with those used in comparable studies and remain translationally relevant, while being purposefully high to assess the consequences of DDX3X overexpression and the potential toxicity associated with high-dose injections.[9–12] Surprisingly, robust and rapid toxicity was observed, where all males and females injected with AAV9/CBA-DDX3X at 2.6×10^{11} vg died abruptly by P8, 1 week post injection (Figure 1A). Compared with vehicle treatment, this was significant (p = 0.014 males, p = 0.005 females), and compared with AAV9/CBA-DDX3X at 1.3×10^{11} vg, this was significant (p = 0.019 males, p = 0.005 females). Survival was also affected by DDX3X overexpression at the lower dose, where by P21, 20% of females and 80% of males injected with AAV9/CBA-DDX3X at 1.3×10^{11} vg had died (Figure 1A). Compared with vehicle treatment, this was significant for males (p = 0.026) but not for females (p = 0.317). At this dose, the males trended toward reduced survival compared with females (p = 0.08). Weight was measured every other day, and no significant weight differences between groups were observed, suggesting that death was not due to failure to thrive (data not shown). Additionally, mice that received AAV9/CBA-DDX3X at 2.6×10^{11} vg on P21 all survived to study endpoint 4 weeks post direct-CSF injection via lumbar intrathecal (i.t.) administration (data not shown), which could indicate a higher sensitivity in younger mice, but it is more likely a factor of a higher dose per body weight at the younger age.

To determine cause of death from overexpressing DDX3X, another cohort of male and female wild-type mice was injected with vehicle or AAV9/CBA-DDX3X at 2.6×10^{11} vg on P1 via i.c.v. administration. On P6, before expected death, necropsies were performed to collect major organs for downstream analysis. DDX3X immunohistochemistry (IHC) and RNA biodistribution was performed on heart, liver, and brain tissue to confirm overexpression of DDX3X (Figures 1B, S1A, and S1B). Notably, even though a direct-CSF injection was used, the vector distributed to peripheral organs, leading to high expression of DDX3X in the heart (~30-fold increase by both IHC and RNA biodistribution) and liver (~6-fold increase by IHC and ~4.6-fold increase by RNA biodistribution) (Figure 1B). Importantly the RNA biodistribution values represent average expression across bulk tissue and may underestimate the magnitude of DDX3× expression in individual transduced cells.

Hematoxylin and eosin (H&E) staining of heart tissue revealed focal or multifocal areas of myocardial degeneration in all mice injected with AAV9/CBA-DDX3X, a phenotype absent in the mice injected with vehicle (Figures 1C, 1D, and S2A). Upon veterinary pathologist examination, the extent and severity of myocardial degeneration suggested that these mice died from heart failure. Potential mechanisms underlying myocardial degeneration following DDX3X overexpression were explored by assessing two stress-associated pathways. The first involves DNA damage-inducible transcript 3 (*Ddit3*), which encodes CHOP, a pro-apoptotic transcription factor induced during endoplasmic reticulum (ER) stress and integrated stress response (ISR) activation. The second involves BCL2 interacting protein 3 (*Bnip3*), a mitochondrial stress responsive protein implicated in mitophagy and cell death regulation. No changes in *Bnip3* levels were observed from overexpressing DDX3X (data not shown). However, in males and females, *Ddit3* mRNA was significantly increased in the heart (~14-fold increase) and liver (~5-fold increase) on P6 following P1 i.c.v. AAV9/CBA-DDX3X (Figure 1E). The significant increase in *Ddit3* was also observed by P5 (11-fold increase in heart and 3-fold increase in liver), whereas an AAV9 control vector encoding a different transgene, AAV9/CBh-GLDC, did not elicit this response, suggesting that this cell death pathway is activated by the *DDX3X* transgene and not AAV9. Interestingly, P21 i.t. AAV9/CBA-DDX3X treatment can significantly increase *Ddit3* levels in the heart (1.3-fold increase); however, the level of increase compared with the injection on P1 is much smaller. DDX3X regulates RNA metabolism and stress granule biology, which is often tied to ISR activation, which can induce CHOP.[13,14] Additionally, DDX3X has been shown to associate with the *Ddit3* promoter to promote *Ddit3* transcription under ER stress.[15] IHC for cleaved caspase-3 revealed increased positive staining in heart tissue after P1 i.c.v. AAV9/CBA-DDX3X treatment relative to vehicle controls, further suggesting that overexpression of DDX3X leads to stress-induced apoptosis in the heart (Figures 1F, S3A, and S3B).

Overexpressing DDX3X can cause lipid accumulation and hepatic toxicity

An incidental finding from H&E staining of the liver revealed hepatocyte ballooning and clear round vacuoles in all mice injected with P1 i.c.v. AAV9/CBA-DDX3X (Figures 1C, 1D, and S2B). This pathology indicates cell injury typically linked to excessive lipid accumulation, i.e., steatosis, and conditions like nonalcoholic fatty liver disease (NAFLD) and nonalcoholic steatohepatitis (NASH).[16] Serum analysis of P6 male and female wild-type mice injected with AAV9/CBA-DDX3X at 2.6×10^{11} vg on P1 revealed a significant 2.4-fold

staining of heart and liver sections scanned at 20× magnification from representative wild-type mice on P6 following an i.c.v. injection of vehicle or AAV9/CBA-DDX3X (2.6×10^{11} vg) on P1. Arrow indicates area of myocardial degeneration. Scale bars represent 100 μm. (D) Summary table of pathological findings from H&E staining of heart, liver, brain, and kidney sections from wild-type mice on P6 following an i.c.v. injection of vehicle or AAV9/CBA-DDX3X (2.6×10^{11} vg) on P1. (E) Relative *Ddit3* mRNA expression in the heart, liver, and brain from female and male mice injected with vehicle or AAV9/CBA-DDX3X (2.6×10^{11} vg) on P1 or P21 via a direct-CSF injection. Relative expression differences were assessed by pairwise Welch's *t* tests on log2FC for each group compared with the female wild-type vehicle group with Benjamini-Hochberg adjustment for multiple testing. Error bars represent standard error of the mean. (F) Cleaved caspase-3 IHC staining of heart sections scanned at 40× magnification from representative wild-type mice on P6 following an i.c.v. injection of vehicle or AAV9/CBA-DDX3X (2.6×10^{11} vg) on P1. Scale bars represent 50 μm.

increase in triglyceride levels, along with a significant 4.2-fold and 2.3-fold increase in aspartate aminotransferase (AST) and alanine aminotransferase (ALT), respectively (Figure 2A). AST can rise with muscle injury, but together with ALT elevation and no change in creatine kinase levels, the results are more consistent with liver injury, common in NAFLD and hepatitis.

To confirm steatosis, oil red O (ORO) staining of heart, liver, brain, and kidney sections from male and female mice on P6 that received P1 i.c.v. AAV9/CBA-DDX3X or vehicle was performed. As expected for nursing pups, baseline lipid droplet signal was detected in the liver and heart of vehicle-treated controls (Figures 2B, 2C, S4A, S4B, S5A, and S5B). Overexpression of DDX3X in the liver caused clear hepatic lipidosis, where all mice showed diffuse micro- and macro-vesicular steatosis from overexpression of DDX3X compared with scattered small to medium size droplets in vehicle controls (Figures 2B, 2C, S4A, and S4B). This phenotype was not as notable in treated mice that were taken down just 1 day prior on P5 (Table S1). In the heart, more subtle but clear changes were noted, where the scattered very small lipid droplets increased in size after P1 i.c.v. AAV9/CBA-DDX3X, but they were still scattered (Figures 2B, 2C, S5A, and S5B). Notably lipid droplets were not detected in the brain or kidney (Figures 2B and 2C), and in a previous experiment where ORO staining was performed on heart, liver, and lung tissue from female mice that received P1 AAV9/CBA-DDX3X via direct-CSF injection, lipid droplets were not detected in the lung (Table S1). This tissue-restricted phenotype, where ORO staining showed clearly elevated lipid droplets in liver and heart, but not brain, kidney, or lung, suggests that DDX3X overexpression does not elicit a uniform lipid accumulation response across organs, but instead, it results in tissue-specific susceptibility for altered lipid handling at this developmental age.

To further support the finding that DDX3X overexpression leads to steatosis, core lipid handling genes, including perilipin 2 (*Plin2*), peroxisome proliferator activated receptor alpha (*Pparα*), apolipoprotein B (*ApoB*), CD36 molecule (*Cd36*), and carnitine palmitoyltransferase 1A (*Cpt1a*) were assessed. Notably, *Plin2*, which encodes the lipid droplet coat protein used as a marker for lipid droplet formation, was significantly increased in the liver of mice that received P1 i.c.v. AAV9/CBA-DDX3X compared with vehicle, with higher levels on P6 (∼4-fold increase) than on P5 (2.5-fold increase), aligning with the ORO staining results (Figure 2D). This was also seen in the heart: however, only at P6 (∼3-fold increase). *Plin2* was not increased in mice that received P21 i.t. AAV9/CBA-DDX3X. *Pparα* encodes a transcription factor that acts as a master regulator controlling fatty acid oxidation and inflammation. On P6, female mice treated with P1 i.c.v. AAV9/CBA-DDX3X had significantly reduced *Pparα* mRNA levels in the liver (2.5-fold decrease) compared with vehicle controls (Figure 2E). A trend toward reduced *Pparα* mRNA levels in the liver from DDX3X overexpression was seen on P5 (females) and P6 (males). However, no changes in *Ppara* levels were seen in the heart. Additionally, no changes in *ApoB*, *Cd36*, or *Cpt1a* levels in the liver were observed by any treatment condition (Figure S4C). Together these results suggest lipid droplet accumulation is more consistent with altered fatty acid oxidation pathways that lead to liver damage, rather than increased CD36-mediated fatty acid uptake or increased ApoB-mediated lipoprotein export.[17]

Overexpressing DDX3X can activate type I interferon signaling

The RIG-I/MAVS → TBK1/IKKε → IRF3/IRF7 axis is central to innate immune signaling and type I interferon (IFN-I) induction (Figure 3A).[21] In this pathway, DDX3X acts as a positive regulator, by interacting with signaling proteins like MAVS, IKKε, TBK1, and IRF3, to promote IFN-I signaling and downstream interferon stimulated genes and chemokine expression.[18–20,22] Interferon beta 1 (*Ifnb1*), which encodes IFN-β, was significantly increased in the heart of mice that received P1 i.c.v. AAV9/CBA-DDX3X compared with vehicle, with higher levels on P6 (>1000-fold increase) than on P5 (140-fold increase) (Figure 3B). DExD/H-box helicase 58 (*Ddx58*), which encodes the pattern recognition receptor RIG-I, was also significantly increased in the heart of mice that received P1 i.c.v. AAV9/CBA-DDX3X compared with vehicle, with higher levels on P6 (∼9-fold increase) than on P5 (4-fold increase) (Figure 3C). Although RIG-I is classically positioned upstream of IFBN1 induction during viral RNA sensing, it is itself an interferon-stimulated gene, so the results are consistent with an IFN-I-driven positive-feedback loop.

C-X-C motif chemokine ligand 10 (*Cxcl10*) is another interferon-stimulated gene and chemokine that recruits immune cells during inflammation. *Cxcl10* was significantly increased in the heart of mice that received P1 i.c.v. AAV9/CBA-DDX3X compared with vehicle, with higher levels on P6 (∼220-fold increase) than on P5 (61-fold increase) (Figure 3D). Interestingly *Cxcl10* mRNA levels were also increased in the brain (23-fold increase), suggesting a direct pathway for *Cxcl10* activation independent of IFN-I signaling due to DDX3X overexpression; however, no known direct link between DDX3X and CXCL10 has been published.

Importantly, even though RIG-I has been shown to interact with AAV and trigger IFN-β activation,[23] our data do not support AAV9 capsid-mediated immune activation as the control AAV9/CBh-GLDC vector did not elicit increased *Ifnb1*, *Ddx58*, or *Cxcl10* levels (Figures 3B–3D). Interestingly, a consistent trend for increased *Ifnb1*, *Ddx58*, and *Cxcl10* levels in males compared with females injected with AAV9/CBA-DDX3X was observed in both the heart and liver (Figures 3B–3D), which may hint at sex-specific sensitivity to DDX3X dosage and could tie back to the survival results from low-dose injection where males were dying before females. Lastly, the IFN-I pathway was not activated by P21 i.t. AAV9/CBA-DDX3X in the heart or liver as no changes in *Ifnb1*, *Ddx58*, or *Cxcl10* levels were observed (Figures 3B–3D), consistent with maintained survival after injection at this age.

DISCUSSION

AAV-mediated gene therapy, while generally safe, has had a long history of recurring toxicity hurdles, attributable to the AAV capsid

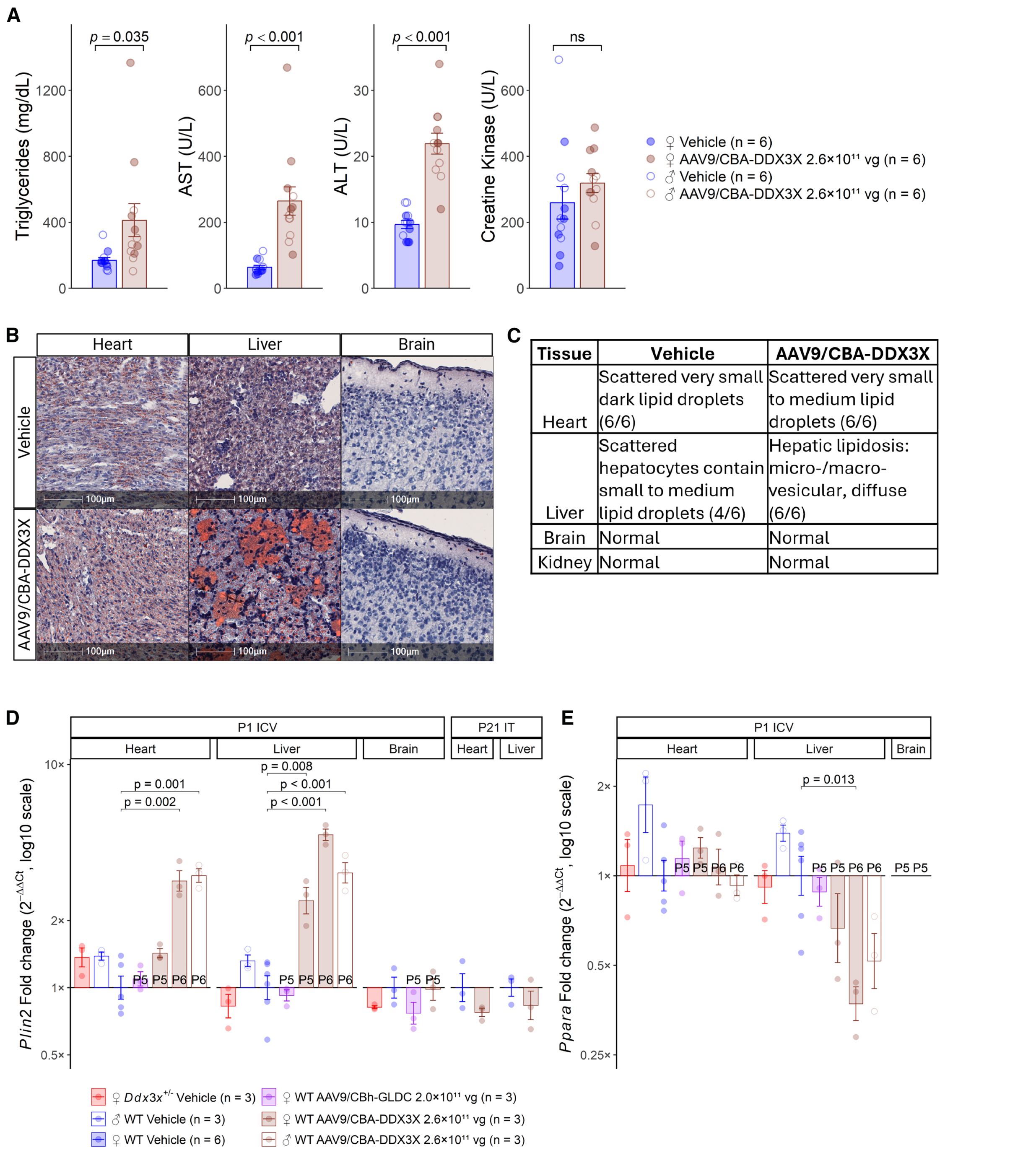

Tissue	Vehicle	AAV9/CBA-DDX3X
Heart	Scattered very small dark lipid droplets (6/6)	Scattered very small to medium lipid droplets (6/6)
Liver	Scattered hepatocytes contain small to medium lipid droplets (4/6)	Hepatic lipidosis: micro-/macro-vesicular, diffuse (6/6)
Brain	Normal	Normal
Kidney	Normal	Normal

Figure 2. Overexpressing DDX3X can cause lipid accumulation and hepatic toxicity

(A) Triglyceride, aspartate aminotransferase (AST), alanine aminotransferase (ALT), and creatine kinase levels in serum from wild-type mice on P6 following an i.c.v. injection of vehicle or AAV9/CBA-DDX3X (2.6×10^{11} vg) on P1. Statistical comparisons between groups were calculated using pairwise Welch's *t* tests. Error bars represent standard error of the mean. (B) Oil red O staining of heart, liver, and brain sections scanned at 20× magnification from representative wild-type mice on P6 following an i.c.v. injection of vehicle or AAV9/CBA-DDX3X (2.6×10^{11} vg) on P1. Scale bars represent 100 μm. (C) Summary table of pathological findings from oil red O staining in heart, liver, brain, and kidney sections from wild-type mice on P6 following an i.c.v. injection of vehicle or AAV9/CBA-DDX3X (2.6×10^{11} vg) on P1. Relative (D) *Plin2* and (E) *Ppara* mRNA expression in the heart, liver, and brain from female and male mice injected with vehicle or AAV9/CBA-DDX3X (2.6×10^{11} vg) on P1 or P21 via a direct-CSF injection. Relative expression differences were assessed by pairwise Welch's *t* tests on log2FC for each group compared with the female wild-type vehicle group with Benjamini-Hochberg adjustment for multiple testing. Error bars represent standard error of the mean.

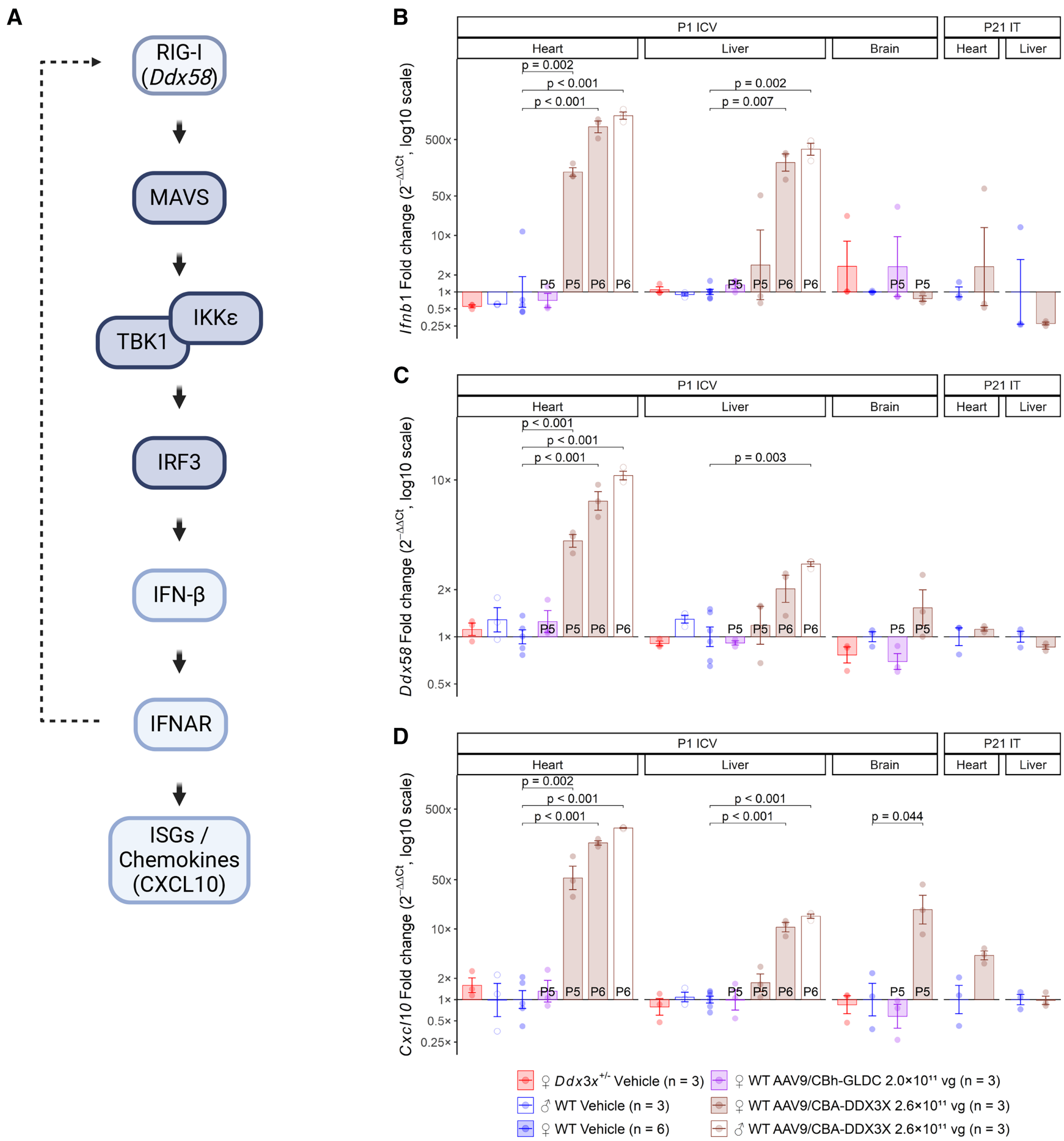

Figure 3. Overexpressing DDX3X can activate the type I interferon pathway

(A) Simplified schematic of type I interferon signaling mediated by RIG-I (*Ddx58*). Factors in dark blue denote reported direct interaction partners of DDX3X that promote signaling, positively regulating type I interferon induction. Factors in light blue have no reported direct interaction with DDX3X.[18–20] RIG-I is also an interferon-stimulated gene (ISG); therefore, type I interferon signaling can increase RIG-I, creating a positive-feedback loop. Relative (B) *Ddx58*, (C) *Ifnb1*, and (D) *Cxcl10* mRNA expression in the heart, liver, and brain from female and male mice injected with vehicle or AAV9/CBA-DDX3X (2.6 × 10^{11} vg) on P1 or P21 via a direct-CSF injection. Relative expression differences were assessed by pairwise Welch's *t* tests on log2FC for each group compared with the female wild-type vehicle group with Benjamini-Hochberg adjustment for multiple testing. Error bars represent standard error of the mean.

and/or packaged transgene.[24,25] Immune responses can be activated against the capsid by pre-existing AAV-specific antibodies, complement activation, and innate and adaptive immune signaling. Liver toxicity, for example, is a common significant adverse event where immune activation is a major driver, with severity ranging from mildly elevated ALT and AST to acute liver failure in extreme cases.[26] However, instead of an immune response to the AAV9 capsid, our data point to a transgene-triggered immune response, where DDX3X overexpression activates IFN-I pathway, particularly in the heart and liver.

While not mechanistically addressed in this paper, IFN-I signaling can lead to *Ddit3*/CHOP activation by pushing cells into ER stress

and inducing an ISR. If increased *Ddit3* is responsible for the caspase-3-dependent apoptosis observed in the heart, this could be a reasonable mechanism leading to myocardial degeneration. Given that DDX3X affects the expression of many genes, there are likely many other targets that could have contributed to the toxicities that were observed. We speculate that some of the factors we explored, such as *Cxcl10* and *Ddit3*/CHOP, can even be modulated through direct interactions with DDX3X. Other factors could have been elevated through primary interactions with DDX3X or as downstream effects further complicating the mechanism leading to death.

Transgene-driven toxicity is typically related to expression levels, including off-target expression and unregulated expression based on the expression cassette design. Consistent with this, overt toxicity was not observed when DDX3X expression was driven by a less potent ubiquitous promoter.[8] Additionally, a recent publication demonstrated that during AAV gene transfer, heart toxicity can be induced by the transgene, aligning with multiple reports of myocarditis in treated patients.[27] In that study, mice were injected systemically at 4 weeks of age with AAV vectors encoding one of three therapeutic transgenes for muscle disease. High doses, resulting in overexpression of the transgene in the heart, caused heart toxicity across all three transgenes with varied severity and mechanisms, whereas an empty AAV vector did not induce this pathology. While our study has some notable differences, including direct-CSF administration, younger age of injection, and not a muscle disease gene, our data support that the heart is particularly sensitive to transgene expression levels. Further support of this is another study that utilized a direct-CSF delivery approach of AAV9-mediated gene supplementation and reported complication in the heart.[28] The vector used in that study also used a strong ubiquitous promoter. It is also worth mentioning that AAV9 efficiently transduces cardiac tissue.

Our results suggest that regulation of DDX3X in certain cell and tissue types will be critical for gene therapy safety. While heart toxicity is the predominant concern, the incidental finding of DDX3X-induced steatosis was notable. Interestingly, patients with NAFLD and NASH have reduced hepatic DDX3X expression, and experimental reduction of DDX3X in hepatocytes exacerbates the fatty liver disease phenotypes.[29,30] This is not the only context where DDX3X has been shown to be implicated in lipid handling and lipid homeostasis[31,32]; however, to our knowledge, this is the first showing that steatosis can also be induced by DDX3X overexpression. It is plausible that lipotoxicity in combination with IFN-I activation in the heart and liver synergize to drive ER stress and the ISR, creating a feedforward loop that accelerates cellular injury and causes rapid death. Considering the results from this study, we raise caution to the use of strong promoters like CBA and regulatory elements such as the woodchuck hepatitis virus posttranscriptional regulatory element to boost transgene expression, especially for transgenes that are dose sensitive like DDX3X. While AAV-mediated transgene expression is often thought to increase slowly, our study illustrates how severe transgene-related complications including death can occur within days rather than weeks.

MATERIALS AND METHODS

Mice

All procedures were approved by UT Southwestern Medical Center's Institutional Animal Care and Use Committee. Female and male C57BL/6J mice (strain #000664, The Jackson Laboratory) were bred to generate most wild-type mice in this study. Additional mouse line information is included in the supplemental information. The mice were housed in a controlled facility on a 12-h light/dark cycle and provided with 16%–18% protein irradiated chow and water *ad libitum*.

Viral vectors

A single-stranded AAV9 vector expressing a codon-optimized human *DDX3X* transgene (h*DDX3X*opt), driven by the strong ubiquitous chicken β-actin (CBA) promoter, and stopped by a synthetic polyadenylation element, SpA, was designed. The CBA-h*DDX3X*opt-SpA plasmid was cloned and sequence verified using standard techniques and a previously published h*DDX3X*opt-SpA.[8] The vector genome was packaged into AAV9 capsids and formulated in PBS containing 5% D-sorbitol and 0.001% poloxamer 188 at the Translational Gene Therapy Core at UT Southwestern Medical Center. The AAV9/CBh-GLDC vector was manufactured at the Vector Core at University of North Carolina and was chosen as the control vector in this study because it also has a single-stranded genome and drives high transgene expression from a strong promoter. Quality control testing for each viral vector is included in the supplemental information (Figures S6–S8). The vectors were stored at −80°C until use, and thawed aliquots were stored at 4°C. The formulation buffer was used as the vehicle and diluent in all experiments.

Viral vector injections

Mice were injected at P1 or P21 with either vehicle or the viral vector. Injections were CNS directed into the CSF via i.c.v. or i.t. routes of administration. The i.t. injection was performed as previously described.[33] The i.c.v. injection was modified from a previously described protocol,[34] with the full description provided in the supplemental information. The highest dose in the mouse studies (2.6×10^{11} vg) would roughly equate to a human dose of 1×10^{15} vg, if extrapolated by neonatal brain mass (375 g human vs. 0.087 g mouse). Several intra-CSF AAV9 clinical trials utilize a dose of 1×10^{15} vg per patient.

Serum and tissue collection

Serum and tissue were collected on P5 or P6 from mice injected on P1 and at 7 weeks from mice injected on P21. The methods for perfusion, tissue collection, and fixation before histology are described in thesupplemental information. Blood was left to clot at room temperature for 1 h after collection and then centrifuged at 8500 rpm for 15 min at 4°C to separate serum. The serum was transferred to a new tube and stored at −80°C until processing at IDEXX Bio-Analytics. Analytes assessed included aspartate aminotransferase,

alanine aminotransferase, creatine kinase, triglycerides, hemolysis, and the lipemia index.

Quantitative reverse-transcription PCR

Total RNA was purified from the frozen tissue using the miRNeasy Mini Kit (Qiagen, 1038703) following the manufacturers purification of total RNA, including small RNAs, from animal tissues protocol. The disruption and homogenization step was conducted using a TissueLyser LT (Qiagen, 85600), set for 4 min at 50 Hz. All RNA samples were stored at −80°C. Complementary DNA (cDNA) was synthesized from purified RNA using SuperScript IV VILO Master Mix with ezDNase enzyme (11766500, Invitrogen) following the manufacturers reverse transcription protocol. All cDNA samples were stored at −30°C. Quantitative reverse-transcription PCR (RT-qPCR) was performed on a real-time PCR system (QuantStudio 5, Applied Biosystems) using SYBR Green (A25742, Applied Biosystems). Primer sequences were obtained from OriGene Technologies and synthesized by Sigma-Aldrich (Table S2). Undetermined cycle threshold (Ct) values were set to 40 before analysis. Three technical replicates were averaged per sample. When the standard deviation across triplicates exceeded 0.35 Ct, the replicate with the largest absolute deviation from the mean was excluded. For *Ddx3x* biodistribution plots, gene expression was normalized using the ΔCt method and reported as $2^{-\Delta Ct}$, where ΔCt = Ct(Ddx3x) – Ct(Rpl13a). For the remaining gene targets, relative expression was computed using the ΔΔCt method, with the reference control group set to the female wild-type vehicle group. Fold change was reported as 2 ˆ(−ΔΔCt).

Histology

All steps were performed at room temperature unless noted.

IHC

Formalin fixed tissues were processed for paraffin embedding using an automated tissue processor (Excelsior, Thermo Scientific), embedded in paraffin (39602004, Leica) using an embedding station (Leica EG1150H), sectioned at 5 μm using a microtome (Leica RM2235), and mounted onto glass slides. The slides were deparaffinated with xylene and rehydrated with an ethanol gradient. Antigen retrieval was performed using Antigen Unmasking Solution (H-3300, Vector Laboratories). Slides were incubated with 3% hydrogen peroxide for 30 min, washed in 1× PBS for 5 min, and then incubated with 2.5% normal horse serum for 30 min. Slides were then incubated with rabbit anti-DDX3 polyclonal antibody (A300-474A, Bethyl Laboratories) or rabbit anti-cleaved caspase-3 polyclonal antibody (25128-1-AP, Proteintech) diluted 1:500 in normal horse serum at 4°C overnight. After washing in distilled water and 1× PBS, slides were incubated with biotinylated anti-rabbit secondary antibody (BA-1000) diluted 1:200 for 1 h. Slides were washed and incubated with ABC kit (PK-6100, Vector Labs) for 30 min and washed with distilled water and 1× PBS. The reaction products were developed with DAB (D4293, Sigma) until the desired stain intensity was achieved, rinsed with distilled water, and counterstained with Modified Mayer's hematoxylin.

H&E

Formalin fixed tissues were embedded in paraffin and sectioned as described in the IHC section. The slides were deparaffinized with xylene and rehydrated with an ethanol gradient and then washed in running tap water for 5 min, followed by incubation in hematoxylin (Richard-Allan Scientific) for 4 min. The slides were then washed in distilled water twice for 10 min each, followed by being dipped in eosin solution for 1 min and dehydrated with an ethanol gradient and cleared with xylene before being mounted with mounting medium.

ORO

Paraformaldehyde fixed tissues embedded in optimal cutting temperature compound were sectioned at 8 μm using a cryostat (Leica CM3050 S) and mounted onto glass slides. The slides were fixed in a 4% paraformaldehyde solution, followed by three deionized water changes. Following fixation, the slides were stained in an ORO working solution containing 0.3% ORO and 1% dextrin. Excess dye was rinsed through three deionized water changes or until the water ran clear. The slides were then counterstained with Hematoxylin 560 (3801575, Leica Biosystems), rinsed in deionized water, and mounted using Fluoromount-G mounting medium.

Following staining, a NanoZoomer S60 (Hamamatsu Photonics) was used for whole-slide imaging, and HALO (Indica Labs) was used for quantitative image analysis.

Statistical analyses

Data were analyzed blind to experimental group. Statistics and plots were generated using custom R scripts. Kaplan-Meier survival curve group differences were evaluated using a global log rank test across all groups followed by pairwise log rank comparisons. For comparisons between two groups, pairwise Welch's *t* tests were performed. For RT-qPCR results with multiple groups, pairwise Welch's *t* tests were performed for each group compared with the female wild-type vehicle group with Benjamini-Hochberg adjustment for multiple testing.

DATA AND CODE AVAILABILITY

The data presented in this study are available on request from the corresponding author.

ACKNOWLEDGMENTS

Research reported in this publication was financially supported by a sponsored research agreement from the DDX3X Foundation to S.J.G. and X.C. and a predoctoral fellowship from the DDX3X Foundation to A.B. Many core facilities at UT Southwestern Medical Center were instrumental in this project including the Transgenic Technology Center Core, Translational Gene Therapy Core Facility, Histo Pathology Core, and Whole Brain Microscopy Facility (RRID: SCR_017949). We also thank the McDermott Center Bioinformatics Lab at UT Southwestern Medical Center for their help analyzing the whole genome sequencing results for our DDX3X mouse model, IDEXX BioAnalytics for performing serum analyte measurements, the Vector Core at the University of North Carolina for manufacturing the viral vector used as the control in this study, and Rachel Adams for providing the *DDX3X* qPCR primers. Select figures were created in BioRender.

AUTHOR CONTRIBUTIONS

Conceptualization, A.B., S.J.G.; investigation, A.B., Y.H., C.L.E., X.C.; formal analysis, A.B., M.W.-C.; visualization, A.B.; writing – original draft preparation, A.B.; writing—

review and editing, A.B., X.C., S.J.G. All authors have read and agreed to the published version of the manuscript.

DECLARATION OF INTERESTS

The authors declare no competing interests.

SUPPLEMENTAL INFORMATION

Supplemental information can be found online at https://doi.org/10.1016/j.ymthe.2026.07.032.

REFERENCES

1. Mo, J., Liang, H., Su, C., Li, P., Chen, J., and Zhang, B. (2021). DDX3X: structure, physiologic functions and cancer. Mol. Cancer *20*, 38. https://doi.org/10.1186/s12943-021-01325-7.
2. Samir, P., and Kanneganti, T.D. (2022). DEAD/H-Box Helicases in Immunity, Inflammation, Cell Differentiation, and Cell Death and Disease. Cells *11*, 1608. https://doi.org/10.3390/cells11101608.
3. Samir, P., Kesavardhana, S., Patmore, D.M., Gingras, S., Malireddi, R.K.S., Karki, R., Guy, C.S., Briard, B., Place, D.E., Bhattacharya, A., et al. (2019). DDX3X acts as a live-or-die checkpoint in stressed cells by regulating NLRP3 inflammasome. Nature *573*, 590–594. https://doi.org/10.1038/s41586-019-1551-2.
4. Sun, M., Zhou, T., Jonasch, E., and Jope, R.S. (2013). DDX3 regulates DNA damage-induced apoptosis and p53 stabilization. Biochim. Biophys. Acta *1833*, 1489–1497. https://doi.org/10.1016/j.bbamcr.2013.02.026.
5. Kwon, J., Choi, H., and Han, C. (2022). A Dual Role of DDX3X in dsRNA-Derived Innate Immune Signaling. Front. Mol. Biosci. *9*, 912727. https://doi.org/10.3389/fmolb.2022.912727.
6. Gadek, M., Sherr, E.H., and Floor, S.N. (2023). The variant landscape and function of DDX3X in cancer and neurodevelopmental disorders. Trends Mol. Med. *29*, 726–739. https://doi.org/10.1016/j.molmed.2023.06.003.
7. Levy, T., Siper, P.M., Lerman, B., Halpern, D., Zweifach, J., Belani, P., Thurm, A., Kleefstra, T., Berry-Kravis, E., Buxbaum, J.D., and Grice, D.E. (2023). DDX3X Syndrome: Summary of Findings and Recommendations for Evaluation and Care. Pediatr. Neurol. *138*, 87–94. https://doi.org/10.1016/j.pediatrneurol.2022.10.009.
8. Boitnott, A., Jiji, A., Plautz, E.J., Hu, Y., Chen, X., and Gray, S.J. (2025). Exploration of a DDX3X Gene Supplementation Therapy Including Expanded Characterization and Novel Findings of Sleep Disturbances in Ddx3x Haploinsufficient Mice. Biol. Psychiatry Glob. Open Sci. *5*, 100599. https://doi.org/10.1016/j.bpsgos.2025.100599.
9. Di Meo, I., Marchet, S., Lamperti, C., Zeviani, M., and Viscomi, C. (2017). AAV9-based gene therapy partially ameliorates the clinical phenotype of a mouse model of Leigh syndrome. Gene Ther. *24*, 661–667. https://doi.org/10.1038/gt.2017.53.
10. Guo, W., Rioux, M., Shaffo, F., Hu, Y., Yu, Z., Xing, C., and Gray, S.J. (2024). AAV9/SLC6A1 gene therapy rescues abnormal EEG patterns and cognitive behavioral deficiencies in Slc6a1-/- mice. J. Clin. Invest. *135*, e182235. https://doi.org/10.1172/JCI182235.
11. Presa, M., Bailey, R.M., Davis, C., Murphy, T., Cook, J., Walls, R., Wilpan, H., Bogdanik, L., Lenk, G.M., Burgess, R.W., et al. (2021). AAV9-mediated FIG4 delivery prolongs life span in Charcot-Marie-Tooth disease type 4J mouse model. J. Clin. Invest. *131*, e137159. https://doi.org/10.1172/JCI137159.
12. Presa, M., Bailey, R.M., Ray, S., Bailey, L., Tata, S., Murphy, T., Piec, P.A., Combs, H., Gray, S.J., and Lutz, C. (2025). Preclinical use of a clinically-relevant scAAV9/SUMF1 vector for the treatment of multiple sulfatase deficiency. Commun. Med. *5*, 29. https://doi.org/10.1038/s43856-025-00734-9.
13. Pakos-Zebrucka, K., Koryga, I., Mnich, K., Ljujic, M., Samali, A., and Gorman, A.M. (2016). The integrated stress response. EMBO Rep. *17*, 1374–1395. https://doi.org/10.15252/embr.201642195.
14. Ryan, C.S., and Schröder, M. (2022). The human DEAD-box helicase DDX3X as a regulator of mRNA translation. Front. Cell Dev. Biol. *10*, 1033684. https://doi.org/10.3389/fcell.2022.1033684.
15. Fan, Z., Xu, L., Gao, Y., Cao, Y., Tian, Y., Pan, Z., Wei, L., Chen, S., Zhang, X., Liu, M., and Ren, F. (2024). The cytoplasmic-nuclear transport of DDX3X promotes immune-mediated liver injury in mice regulated by endoplasmic reticulum stress. Cell Death Dis. *15*, 702. https://doi.org/10.1038/s41419-024-07076-9.
16. Takahashi, Y., and Fukusato, T. (2014). Histopathology of nonalcoholic fatty liver disease/nonalcoholic steatohepatitis. World J. Gastroenterol. *20*, 15539–15548. https://doi.org/10.3748/wjg.v20.i42.15539.
17. Ipsen, D.H., Lykkesfeldt, J., and Tveden-Nyborg, P. (2018). Molecular mechanisms of hepatic lipid accumulation in non-alcoholic fatty liver disease. Cell. Mol. Life Sci. *75*, 3313–3327. https://doi.org/10.1007/s00018-018-2860-6.
18. Oshiumi, H., Sakai, K., Matsumoto, M., and Seya, T. (2010). DEAD/H BOX 3 (DDX3) helicase binds the RIG-I adaptor IPS-1 to up-regulate IFN-beta-inducing potential. Eur. J. Immunol. *40*, 940–948. https://doi.org/10.1002/eji.200940203.
19. Soulat, D., Bürckstümmer, T., Westermayer, S., Goncalves, A., Bauch, A., Stefanovic, A., Hantschel, O., Bennett, K.L., Decker, T., and Superti-Furga, G. (2008). The DEAD-box helicase DDX3X is a critical component of the TANK-binding kinase 1-dependent innate immune response. EMBO J. *27*, 2135–2146. https://doi.org/10.1038/emboj.2008.126.
20. Saikruang, W., Ang Yan Ping, L., Abe, H., Kasumba, D.M., Kato, H., and Fujita, T. (2022). The RNA helicase DDX3 promotes IFNB transcription via enhancing IRF-3/p300 holocomplex binding to the IFNB promoter. Sci. Rep. *12*, 3967. https://doi.org/10.1038/s41598-022-07876-z.
21. Rehwinkel, J., and Gack, M.U. (2020). RIG-I-like receptors: their regulation and roles in RNA sensing. Nat. Rev. Immunol. *20*, 537–551. https://doi.org/10.1038/s41577-020-0288-3.
22. Gu, L., Fullam, A., Brennan, R., and Schröder, M. (2013). Human DEAD box helicase 3 couples IkappaB kinase epsilon to interferon regulatory factor 3 activation. Mol. Cell. Biol. *33*, 2004–2015. https://doi.org/10.1128/MCB.01603-12.
23. Shao, W., Earley, L.F., Chai, Z., Chen, X., Sun, J., He, T., Deng, M., Hirsch, M.L., Ting, J., Samulski, R.J., and Li, C. (2018). Double-stranded RNA innate immune response activation from long-term adeno-associated virus vector transduction. JCI Insight *3*, e120474. https://doi.org/10.1172/jci.insight.120474.
24. Ertl, H.C.J. (2022). Immunogenicity and toxicity of AAV gene therapy. Front. Immunol. *13*, 975803. https://doi.org/10.3389/fimmu.2022.975803.
25. Vandamme, C., Adjali, O., and Mingozzi, F. (2017). Unraveling the Complex Story of Immune Responses to AAV Vectors Trial After Trial. Hum. Gene Ther. *28*, 1061–1074. https://doi.org/10.1089/hum.2017.150.
26. Jagadisan, B., and Dhawan, A. (2023). Hepatotoxicity in Adeno-Associated Viral Vector Gene Therapy. Curr. Hepatol. Rep. *22*, 276–290. https://doi.org/10.1007/s11901-023-00624-5.
27. Biquand, A., Gicquel, E., Poupiot, J., Faivre, M., Campuzano, S., Bourgeton, T., Le Brun, P.R., Veron, P., Brureau, A., and Richard, I. (2026). Transgene-induced cardiotoxicity in high-dose AAV gene transfer. Mol. Ther. *34*, 2012–2027. https://doi.org/10.1016/j.ymthe.2025.12.042.
28. Ling, Q., Rioux, M., Higgs, H., Hu, Y., Dwyer, S.E., and Gray, S.J. (2025). Improved AAV9-based gene therapy design for SURF1-related Leigh syndrome with minimal toxicity. Mol. Ther. Methods Clin. Dev. *33*, 101554. https://doi.org/10.1016/j.omtm.2025.101554.
29. Liu, P., Zhang, Y., Tang, C., Cen, L., Chen, Y., Li, S., Chen, X., Yu, M., Zhang, J., Zhang, X., et al. (2022). The DEAD-box helicase DDX3x ameliorates non-alcoholic fatty liver disease via mTORC1 signalling pathway. Liver Int. *42*, 1793–1802. https://doi.org/10.1111/liv.15278.
30. Yang, S., Zhou, L., Zhao, T., Zhu, H., Luo, T., Jiang, K., Shi, X., Chen, C., Zhang, H., Zhao, S., et al. (2023). Protective and Adverse Roles of DDX3X in Different Cell Types in Nonalcoholic Steatohepatitis Progression. Research (Wash D C) *6*, 0275. https://doi.org/10.34133/research.0275.
31. Dai, J.Z., Hsu, W.J., Lin, M.H., Shueng, P.W., Lee, C.C., Yang, C.C., and Lin, C.W. (2025). YAP-mediated DDX3X confers resistance to ferroptosis in breast cancer cells

by reducing lipid peroxidation. Free Radic. Biol. Med. *232*, 330–339. https://doi.org/10.1016/j.freeradbiomed.2025.03.019.

32. Tsai, T.Y., Wang, W.T., Li, H.K., Chen, W.J., Tsai, Y.H., Chao, C.H., and Wu Lee, Y.H. (2017). RNA helicase DDX3 maintains lipid homeostasis through upregulation of the microsomal triglyceride transfer protein by interacting with HNF4 and SHP. Sci. Rep. *7*, 41452. https://doi.org/10.1038/srep41452.

33. Boitnott, A., Rioux, M., Chen, X., and Gray, S. (2025). Lumbar Intrathecal Injection of Gene Therapy Vectors for Central Nervous System Targeting in Mice and Rats. J. Vis. Exp. https://doi.org/10.3791/67138.

34. Glascock, J.J., Osman, E.Y., Coady, T.H., Rose, F.F., Shababi, M., and Lorson, C.L. (2011). Delivery of therapeutic agents through intracerebroventricular (ICV) and intravenous (IV) injection in mice. J. Vis. Exp. 2968. https://doi.org/10.3791/2968.

YMTHE, Volume *34*

Supplemental Information

DDX3X overexpression in mice can cause rapid tissue-specific toxicity and mortality

Andrea Boitnott, Yuhui Hu, Mary Wight-Carter, Christian Lopez Escobar, Xin Chen, and Steven J. Gray

Supplemental Material

Mice

In addition to the C57BL/6J wild-type mice, a novel loss-of-function DDX3X mouse line was generated by the Transgenic Technology Center Core at UT Southwestern Medical Center. This mouse was used for the RT-qPCR experiments to assess gene expression. It was designed to knock-in an allele containing an invertible stop cassette, configured as a flip-excision (FLEx) switch (FRT-LoxP-Lox2272-inverted SV40pA x3-LoxP-Lox2272-FRT), inserted into *Ddx3x* intron 1. Correct editing was confirmed in the founder by whole-genome sequencing. Mice were bred to obtain *Ddx3x*$^{FLEx/FLEx}$ females, which were then crossed with Sox2-cre/+ males (#008454; The Jackson Laboratory) to generate *Ddx3x*$^{FLEx/+}$ control, referred to as wild-type, and *Ddx3x*$^{stop/+}$ mutant, referred to as *Ddx3x*$^{+/-}$, female littermates. This line was maintained on a C57BL6/J background. Stop cassette efficiency was validated by DDX3X protein quantification via western blot from cortical tissue (data not shown). Additionally, *Ddx3x*$^{+/-}$ females exhibit a phenotype reminiscent of a previously characterized haploinsufficient *Ddx3x* mouse model and of DDX3X syndrome, supporting the relevance and validity of this model (data not shown).

Viral Vector Injections

For bilateral ICV injections, pups were cryoanesthetized and moved to a precooled clay mold for stabilization. A stereotaxic injection syringe (65460-06, Hamilton) with a 33 gauge needle (65461-02, Hamilton) set to a stop depth of 2 mm was used and needle placement was determined based on lambda, the midline, and the eye, such that if a line was drawn from lambda to the eye, the placement would be 1 mm from lambda towards the eye and about 0.5 mm from midline. A light was used to help visualize the skull suture lines under the skin. 1 μL was injected over 1 minute per hemisphere, with a subsequent 30 second hold before withdrawing the needle. Pups were paw tattooed for identification and placed on a heating pad until fully conscious before returning to their home cage.

Tissue Collection

Pups were cryoanesthetized, whereas adult mice were anesthetized with 500 mg/kg 2,2,2-tribromoethanol (421430500, Thermo Scientific Chemicals) dissolved in 2-methyl-2-butanol (152463, Sigma-Aldrich) and diluted in sterile water to a final concentration of 2.5%, prepared fresh on the day of use. Following full anesthesia, the chest cavity was opened and blood was collected by cardiac puncture using a 1 mL syringe fitted with a 25 gauge needle. The mice were then perfused with 1x PBS and 1 U/mL heparin at a flow rate of 1 mL/minute for pups and 3.5 mL/minute for adults. The color of the liver was noted before and after perfusion for necropsies on P6. Heart, liver, lung, kidney, and brain tissue were collected in three ways for downstream processing. (1) Frozen tissue: the outer half of the brain left hemisphere, bottom third or heart, one lobe of the liver, and half of the right kidney cut longitudinally was frozen on dry ice and stored at -80°C. (2) Formalin fixed tissue: the inner half of the brain left hemisphere, upper third of the heart, one lobe of the liver, and half of the right kidney cut longitudinally were fixed in 10% formalin for 24 hours at room temperature. Following fixation, the samples were washed in 70% ethanol three times and then secured in cassettes for paraffin embedding. (3) Paraformaldehyde (PFA) fixed tissue: the brain right hemisphere, middle third of the heart, one lobe of liver, and left kidney were fixed in 4% PFA for 24

hours at 4°C. The samples were then rinsed in Milli-Q water three times, transferred to 10% sucrose + 0.5% sodium azide for 24 hours at 4°C, followed by 18% sucrose + 0.05% sodium azide for 48 hours at 4°C. For brain, an additional sucrose step of 30% sucrose + 0.05% sodium azide for 48 hours at 4°C was done. Following the sucrose gradient, the samples were embedded in optimal cutting temperature (OCT) compound.

Table S1. Pathology report provided by a veterinary pathologist with toxicity findings from H&E and ORO slides, including mouse ID, sex, treatment, route of administration, age at necropsy, and tissue type.

Table S2. Forward and reverse primer sequences used for RT-qPCR.

**Category**	**Gene**	**Forward Primer**	**Reverse Primer**
Gene of Interest	Mouse DEAD-box helicase 3 X-linked (*Ddx3x*)	ATGAGTCATGTGGCAGTGGA	CCTTTGCTTGCTGTACTTCC
	Human *DDX3X* opt	CGAAGATGACTGGAGCAAGC	AATTGTTTCCGGTGGCTTCC
Housekeeping normalization	Ribosomal protein L13a (*Rpl13a*)	CTGCTCTCAAGGTTGTTCGGCT	CCTTCCGTTTCTCCTCCAGAGT
Lipid metabolism and transport	Apolipoprotein B (*ApoB*)	GCATGAGTATGCCAATGGTCTCC	CTGGTTGCCATCTGAAGCCATG
	CD36 molecule (*Cd36*)	GGACATTGAGATTCTTTTCCTCTG	GCAAAGGCATTGGCTGGAAGAAC
	Perilipin 2 (*Plin2*)	GACAGGATGGAGGAAAGACTGC	GGTAGTCGTCACCACATCCTTC
	Carnitine palmitoyltransferase 1A (*Cpt1a*)	GGCATAAACGCAGAGCATTCCTG	CAGTGTCCATCCTCTGAGTAGC
	Peroxisome proliferator activated receptor alpha (*Pparα*)	ACCACTACGGAGTTCACGCATG	GAATCTTGCAGCTCCGATCACAC
Type I interferon-driven innate immune response	DExD/H-box helicase 58 (*Ddx58*; RIG-I)	AGCCAAGGATGTCTCCGAGGAA	ACACTGAGCACGCTTTGTGGAC
	Interferon beta 1 (*Ifnb1*)	GCCTTTGCCATCCAAGAGATGC	ACACTGTCTGCTGGTGGAGTTC
	C-X-C motif chemokine ligand 10 (*Cxcl10*)	ATCATCCCTGCGAGCCTATCCT	GACCTTTTTTGGCTAAACGCTTTC
Cell stress and death	DNA damage inducible transcript 3 (*Ddit3*; CHOP)	GGAGGTCCTGTCCTCAGATGAA	GCTCCTCTGTCAGCCAAGCTAG
	BCL2 interacting protein 3 (*Bnip3*)	GCTCCAAGAGTTCTCACTGTGAC	GTTTTTCTCGCCAAAGCTGTGGC

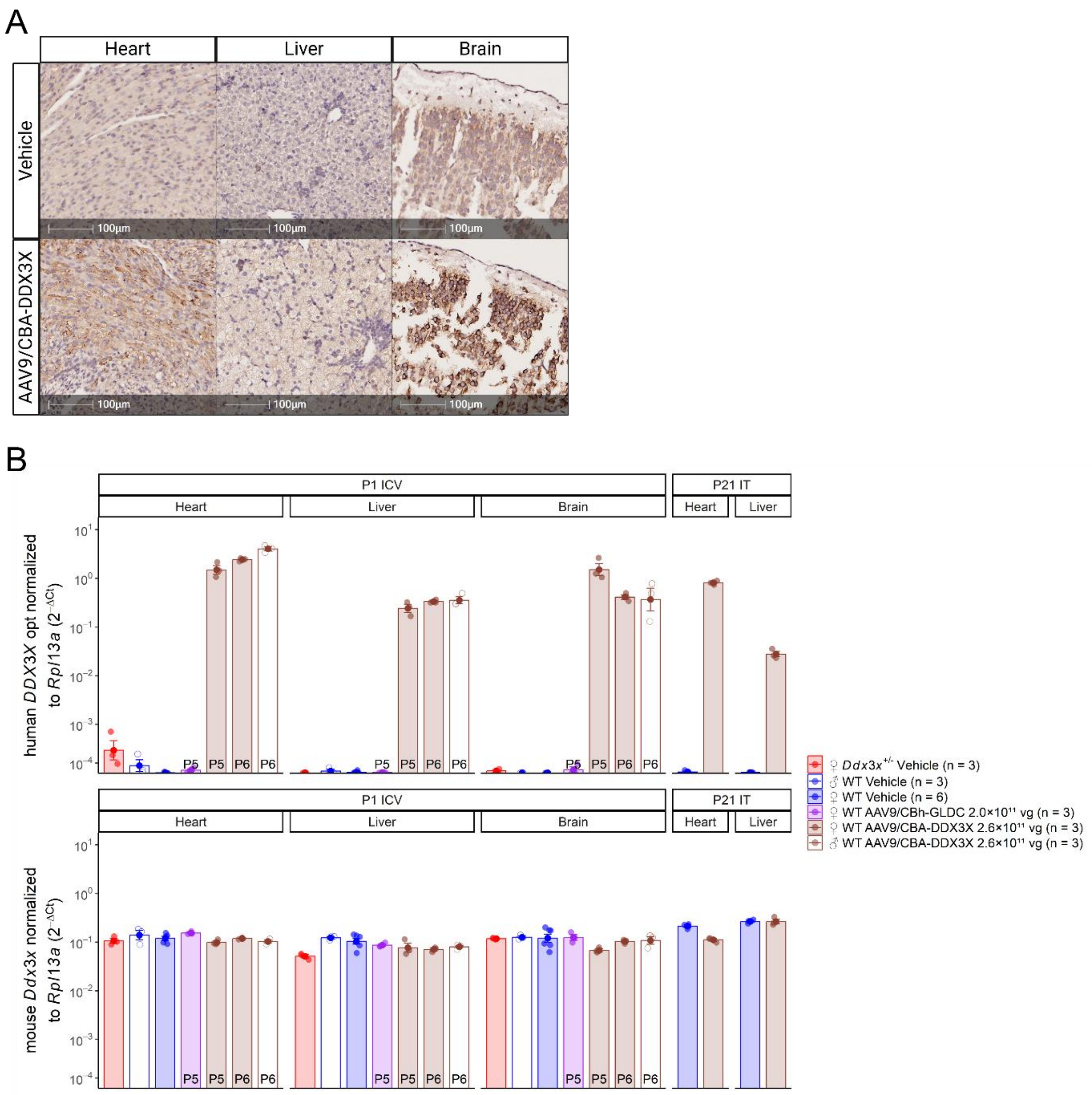

Figure S1. A) DDX3X IHC staining of heart, liver, and brain sections scanned at 20x magnification from representative wild-type mice on P6 following a P1 ICV injection of vehicle or AAV9/CBA-DDX3X (2.6 x 10^{11} vg). Scale bars = 100 µm. Quantification from all mice in Figure 1B. B) Human *DDX3X* opt (top) and endogenous mouse *Ddx3x* (bottom) mRNA expression in the heart, liver, and brain from female and male mice injected with vehicle or AAV9/CBA-DDX3X (2.6 x 10^{11} vg) on P1 or P21 via direct CSF injection. Values are defined as gene expression normalized to the housekeeping gene *Rpl13a*, calculated as $2^{-\Delta Ct}$, where ΔCt = Ct(Ddx3x) – Ct(Rpl13a). A subset is presented in Figure 1B (middle and bottom panels).

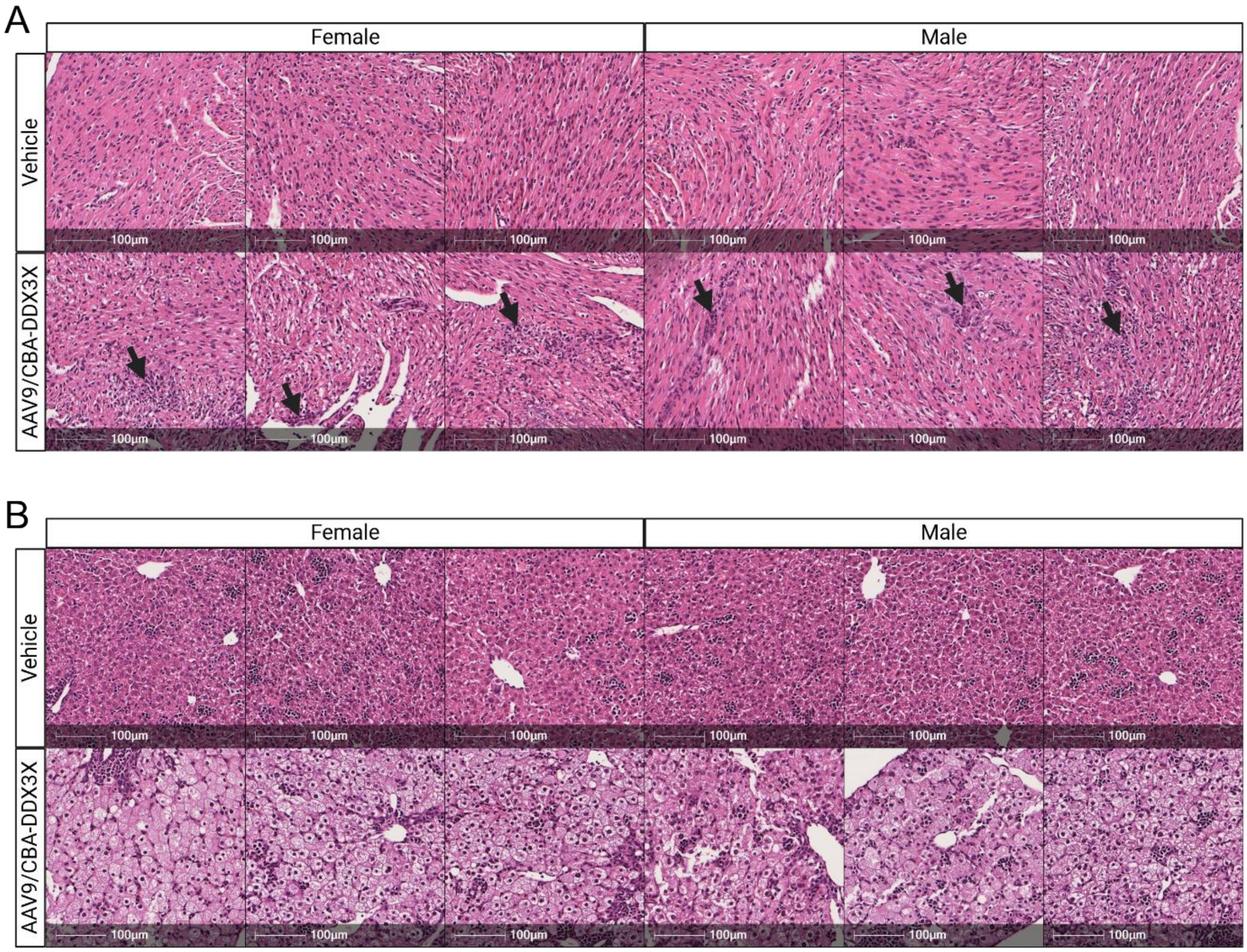

Figure S2. A) H&E staining of heart sections scanned at 20x magnification from wild-type mice on P6 following a P1 ICV injection of vehicle or AAV9/CBA-DDX3X (2.6 x 10^{11} vg). Three biological replicates for each group are shown. Arrows indicate example areas of myocardial degeneration. Scale bars = 100 µm. B) H&E staining of liver sections scanned at 20x magnification from the same mice in A. Column 1 from A and B are used as the representative image in Figure 1C. Scale bars = 100 µm.

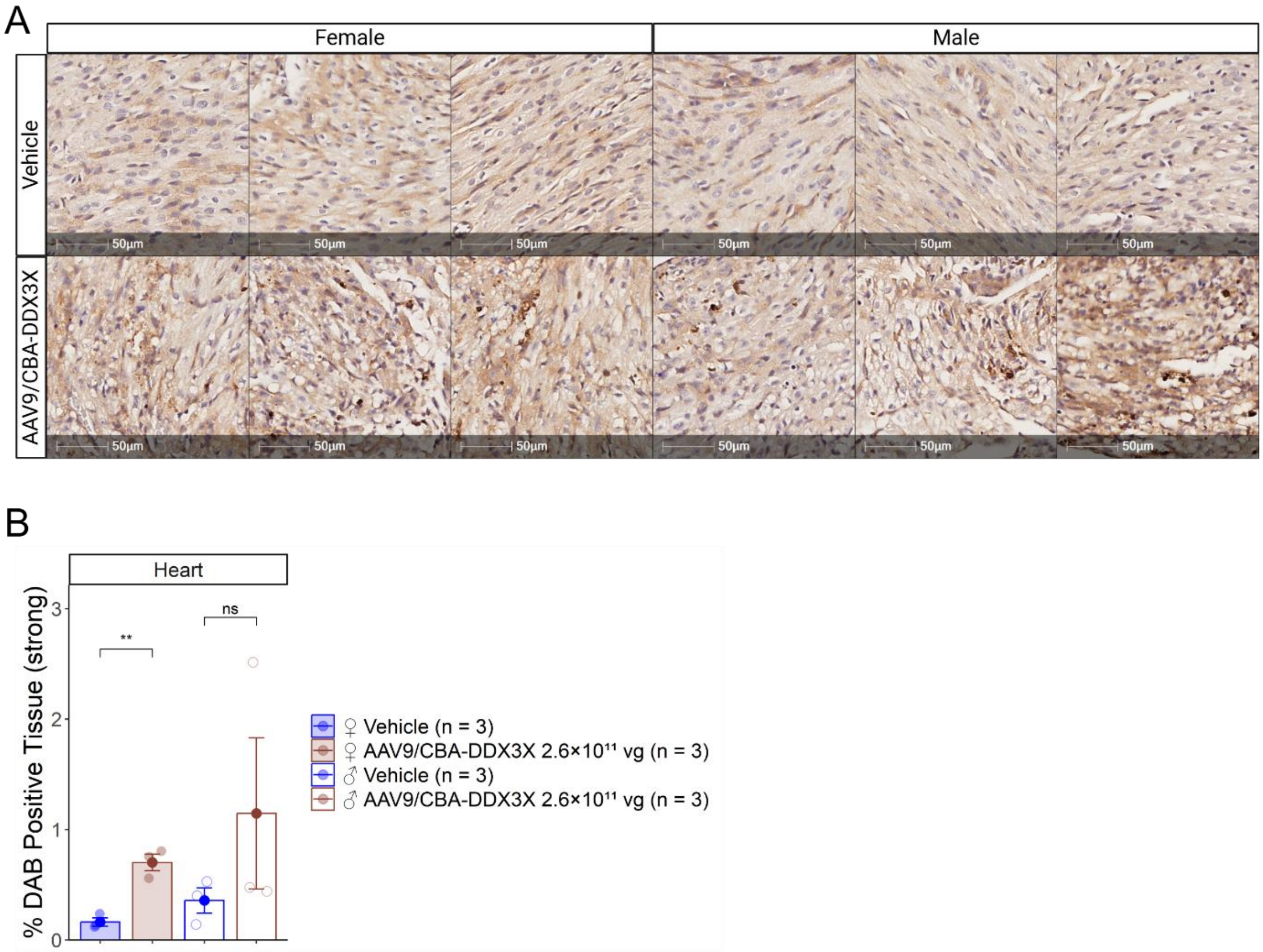

Figure S3. A) Cleaved caspase 3 IHC staining of heart sections scanned at 40x magnification from wild-type mice on P6 following a P1 ICV injection of vehicle or AAV9/CBA-DDX3X (2.6 x 10^{11} vg). Three biological replicates for each group are shown. Column 1 is used as the representative image in Figure 1F. Scale bars = 50 µm. B) Cleaved caspase 3 IHC quantification: Percentage of strong DAB positive stain area in heart sections from the mice with representative images in A. Statistical comparisons between groups were calculated using pairwise Welch's t-tests.

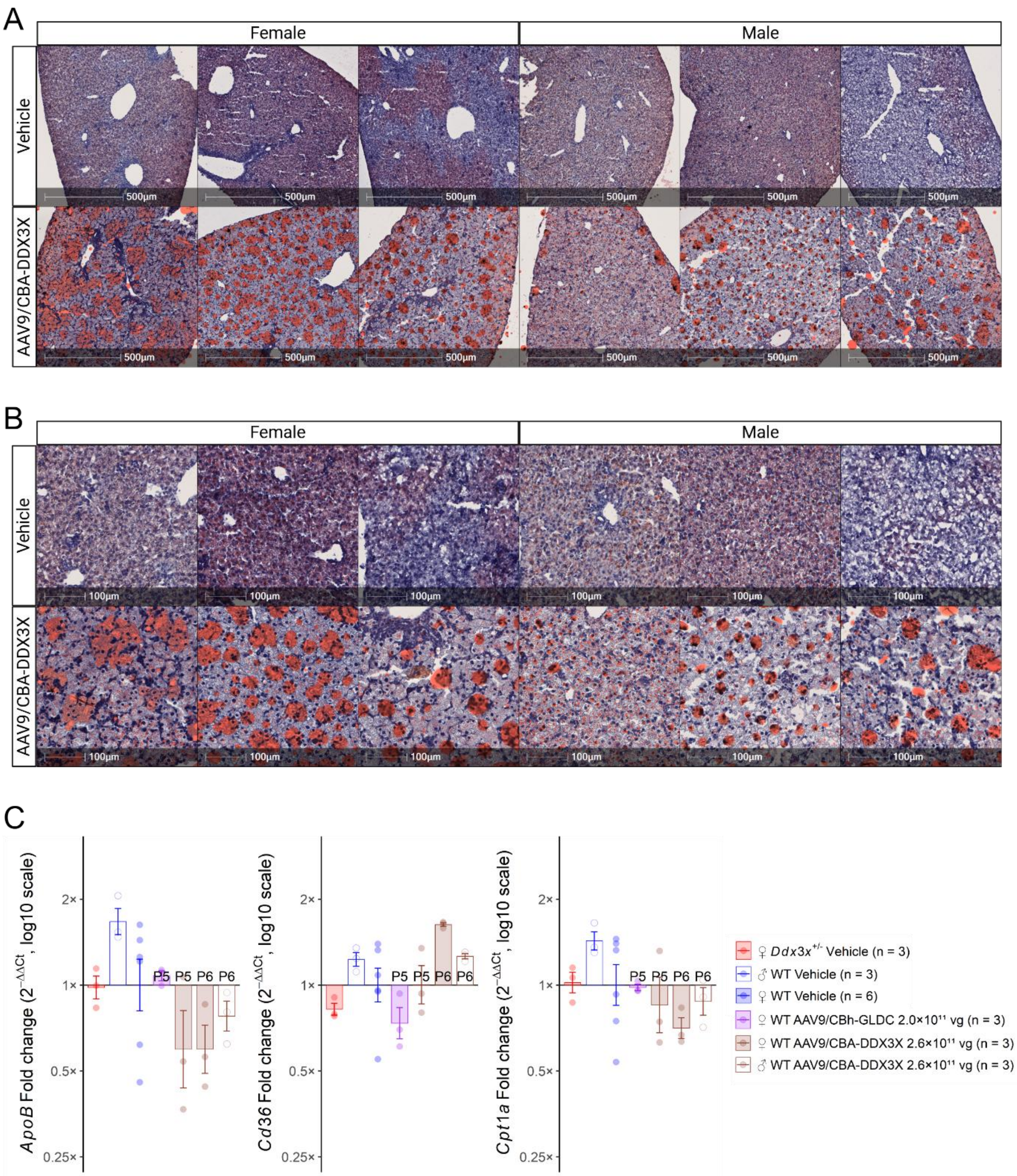

Figure S4. A) ORO staining of liver sections scanned at 7.5x magnification from wild-type mice on P6 following a P1 ICV injection of vehicle or AAV9/CBA-DDX3X (2.6 x 10^{11} vg). Three biological replicates for each group are shown. Scale bars = 500 µm. B) 20x magnification of the sections in A. Column 1 is used as the representative image in Figure 2B. Scale bars = 100 µm. C) Relative *ApoB* (left), *Cd36* (middle), and *Cpt1a* (right) mRNA expression in the liver from female and male mice injected with vehicle or AAV9/CBA-DDX3X (2.6 x 10^{11} vg) on P1 via ICV administration. Relative expression differences were assessed by pairwise Welch's t-tests on log2FC for each group

compared to the female wild-type vehicle group with Benjamini-Hochberg adjustment for multiple testing – no comparison was significant.

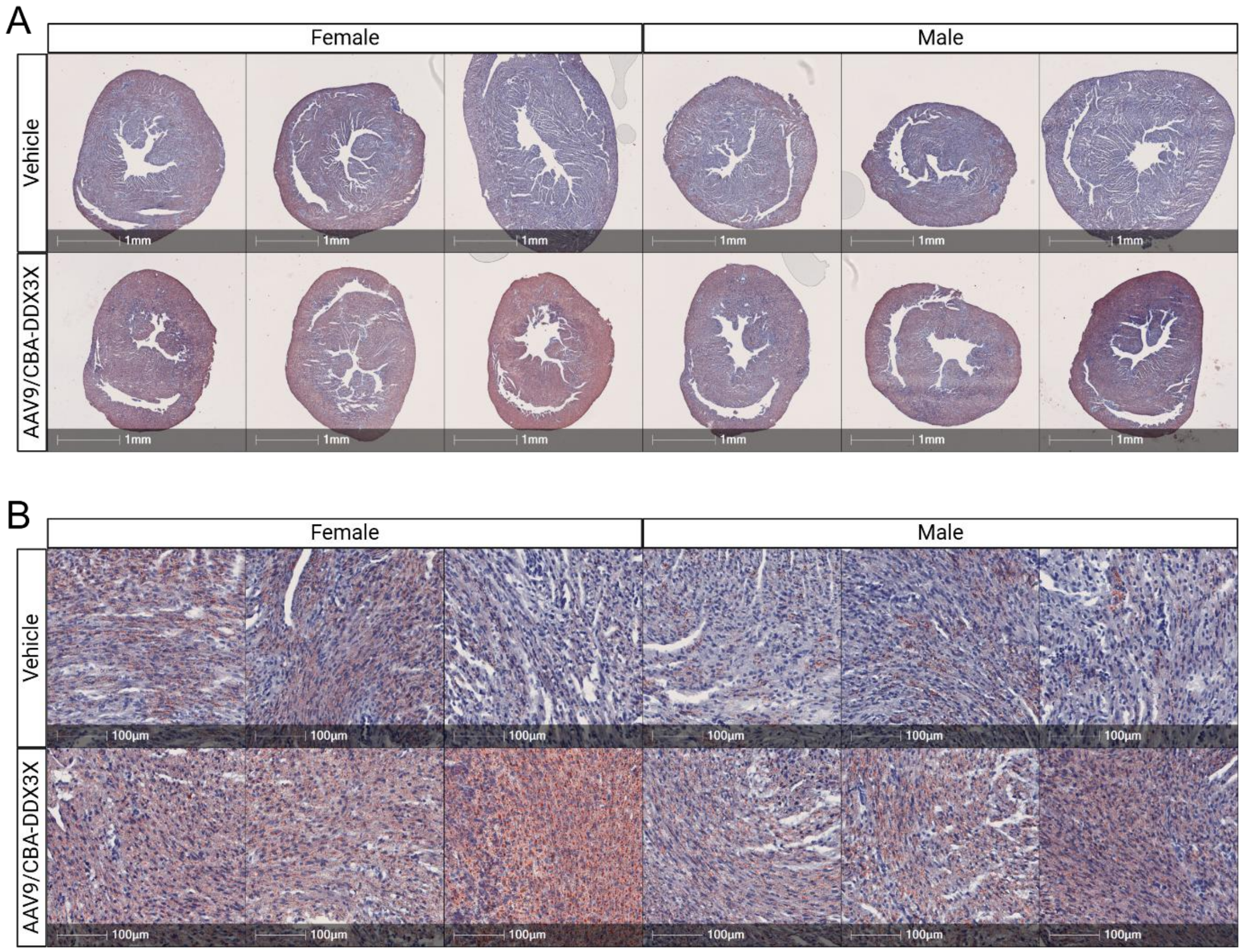

Figure S5. A) ORO staining of heart sections scanned at 2.5x magnification from wild-type mice on P6 following a P1 ICV injection of vehicle or AAV9/CBA-DDX3X (2.6 x 10^{11} vg). Three biological replicates for each group are shown. Scale bars = 1 mm. B) 20x magnification of the sections in A. Column 1 is used as the representative image in Figure 2B. Scale bars = 100 μm.

UT Southwestern
Medical Center

Translational Gene Therapy Core (TGTC)

Certificate of Analysis Form

**Batch ID:**	NC-56-23-001_02
**Product Name:**	pAAV9 / DDX3X
**ITR Plasmid Name:**	pTRsk-CBA-DDX3X-spA
**Manufacturing Date:**	13JUN2023
**Manufacturing Scale**	7.5L (of 10L)
**Vial Number:**	13
**Total Volume:**	0.46mL (10ulx5vials, 50ulx7vials, 60ulx1vial)
**Storage Conditions:**	≤-60°C, Upon thawing, 4°C

Test	Method	Results
Endotoxin	LAL	<1.00 EU/mL
Capsid Identity	ELISA	1.09E+15 Vp/mL
Capsid Identity	Stunner	6.50E+14 Vp/mL
Titer-Vg	qPCR, target ITR	1.78E+14 Vg/mL
Titer-Vg	Stunner	1.44E+14 Vg/mL
Purity	Silver Stain	Detection of VP1, VP2 and VP3 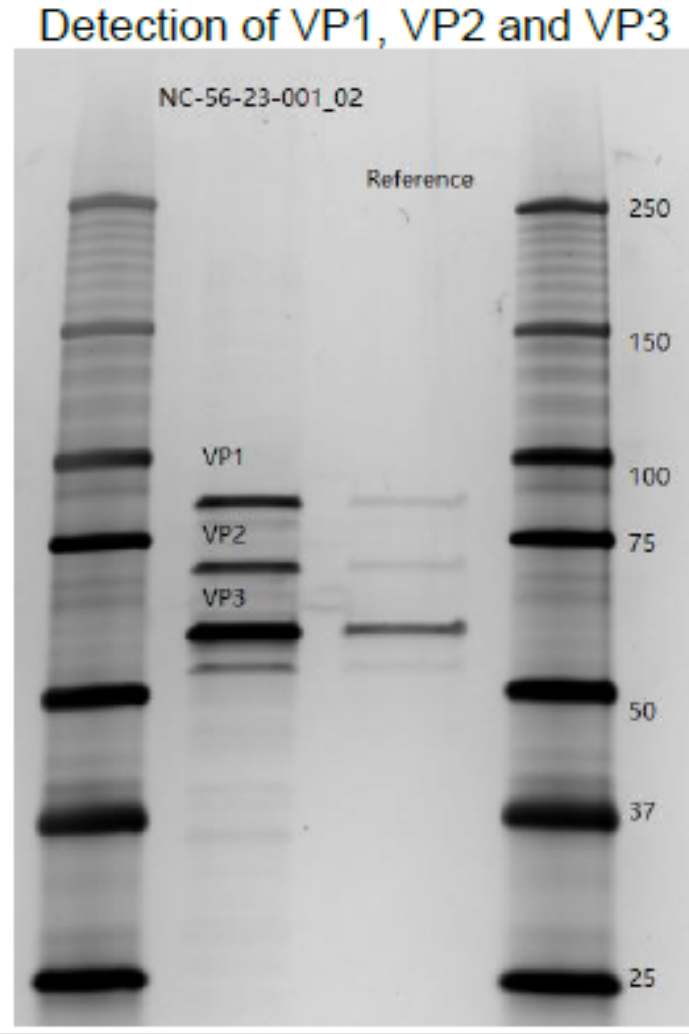

Released By Yang Yu

Digitally signed by Yang Yu
DN: cn=Yang Yu, c=US, o=UTSW, ou=VVC, email=yang.yu@utsouthwestern.edu
Reason: I am the author of this document
Date: 2023.06.16 16:31:51 -05'00'

Figure S6. The certificate of analysis summarizing quality control testing for the manufacture and release of AAV9/CBA-DDX3X from the Translational Gene Therapy Core at UT Southwestern Medical Center. The same batch was used for all injections.

Quality Control Summary

Lot #	LAV117-conc	Name	GLDC

Test by qPCR

Test #	Titer, vg/mL	Analyst	Date	File
1	1.03E+14	PZ	01/29/2020	20200129-1147-gh-pz

PAGE analysis

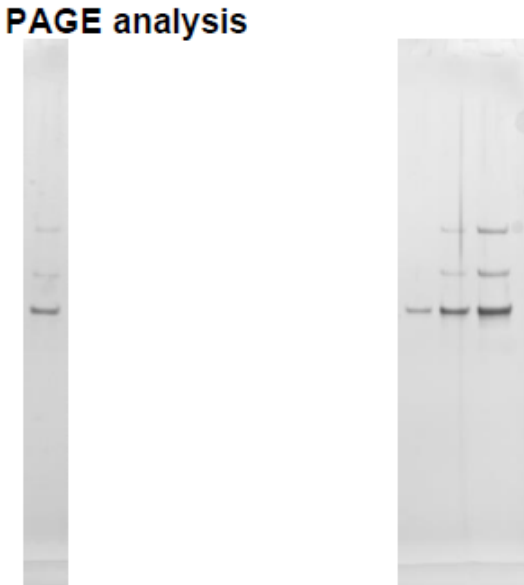

Loaded 5.00E+09 vg 4980E std 2e9vg 5e9vg 1e10vg
Calculated 4.10E+09 vg

Analyst	Ping Zhang
Date	01/13/2020
Reference #	20200113-silver

SEM

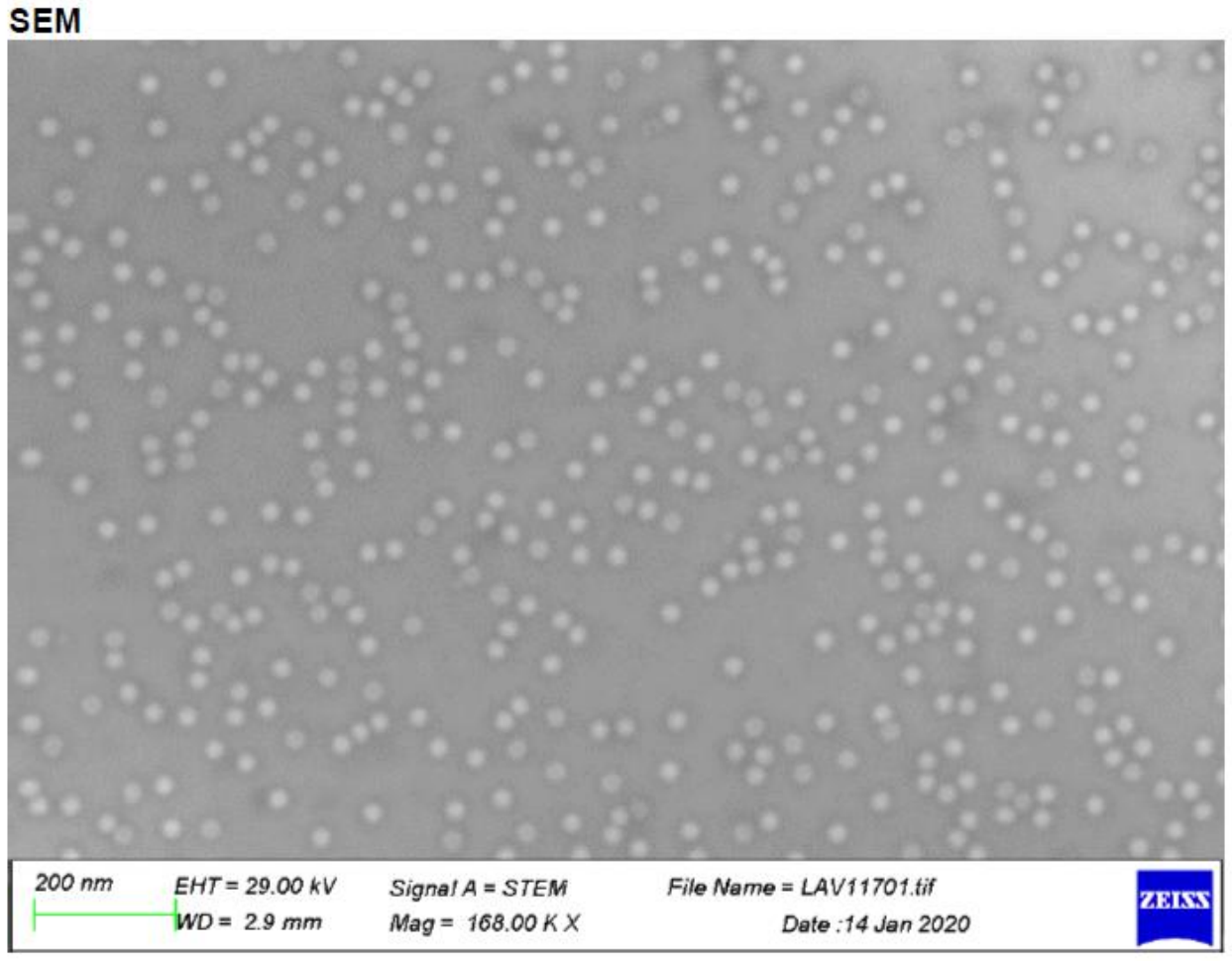

94% full

Analyst	Ping Zhang
Date	12/16/2019
Reference #	20191216-LAV117-01

Figure S7. The certificate of analysis summarizing quality control testing for the manufacture and release of AAV9/CBh-GLDC from the Vector Core at University of North Carolina. The same batch was used for all injections.

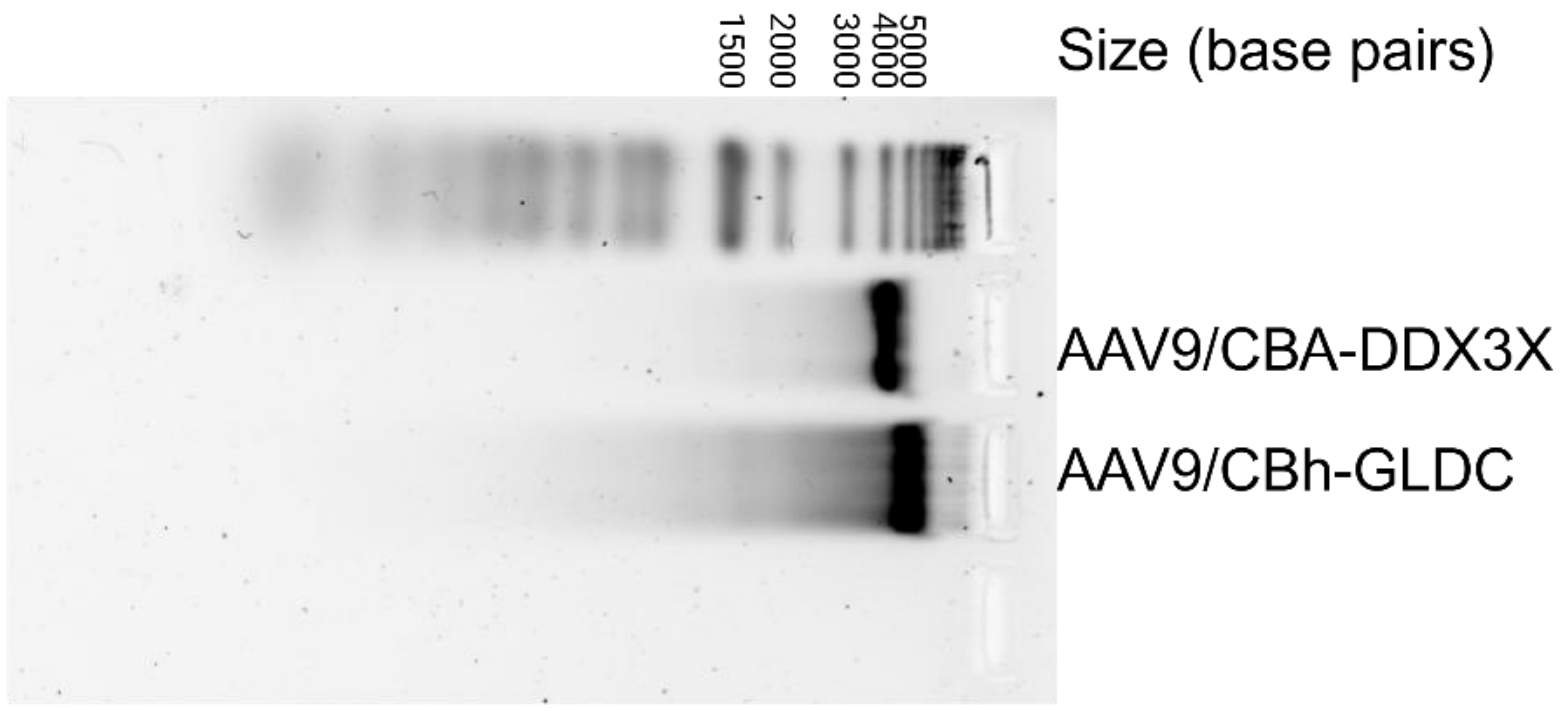

Figure S8. Alkaline gel showing viral vector integrity of AAV9/CBA-DDX3X and AAV9/CBh-GLDC at 5 x 10^{10} vg.
